# A dual functional inhibitory molecule to combat GyrA-ParC and associated mutants in fluoroquinolone-resistant *Shigella flexneri* causing shigellosis: an ML guided and quantum simulation-based *in silico* approach

**DOI:** 10.3389/fbinf.2026.1765216

**Published:** 2026-06-10

**Authors:** Soumyadip Ghosh, Sudha Ramaiah

**Affiliations:** 1 Medical and Biological Computing Laboratory, School of Biosciences and Technology (SBST), Vellore Institute of Technology (VIT), Vellore, India; 2 Department of Bio Sciences, SBST, VIT, Vellore, India

**Keywords:** AMR, ciprofloxacin, fluoroquinolone, machine learning, mutations, QRDR, shigellosis

## Abstract

Shigellosis is a significant threat to global public health, with an estimated 188 million cases resulting in high death rates, with the highest burden among children under 5 years old living in low and middle-income countries. The major treatment options historically have been fluoroquinolones, most commonly ciprofloxacin, and other antibiotics like azithromycin and third-generation cephalosporins. Fluoroquinolone-resistant *S. flexneri* poses an increased risk of causing disease globally and therefore represents a growing public health concern. Fluoroquinolone resistance in *Shigella* is primarily caused by chromosomal mutations in the DNA gyrase and topoisomerase IV subunits, which reduce the affinity of fluoroquinolones for their target sites, thereby reducing the effectiveness of therapy. In this study, we developed a machine-learning guided *in silico* approach to identify a single molecule capable of inhibiting both the DNA gyrase subunit A and topoisomerase IV, including resistant mutants, in *Shigella flexneri*. To accomplish this goal, machine learning models were trained on existing minimum inhibitory concentrations for various active compounds including different antibiotics against *S. flexneri* to screen a library of ciprofloxacin analogues derived from the PubChem database. The top-ranked compounds were then further evaluated utilising a multidisciplinary computational approach that included virtual screening, toxicity assessments, quantum simulations *via* density functional theory, molecular docking studies, molecular dynamic simulations, and binding free energy calculations. The multi-faceted computational workflow identified a lead compound, CA_1617 [PubChem CID: 1350066; IUPAC name: 6-Fluoro-1-(4-fluorophenyl)-7-(4-methyl-3-oxopiperazin-1-yl)-4-oxoquinoline-3-carboxylic acid] that demonstrated a high degree of dual-target affinity for both wild-type and mutant target proteins, had stable protein-ligand interactions, and had favourable pharmacokinetic characteristics relative to the reference antibiotic ciprofloxacin. These results support the potential of the lead molecule CA_1617 as a mutation-resistant inhibitor. However, additional experimental validation is required to determine its efficacy and translate the computational findings of this study into clinical use.

## 1 Introduction

The rise of Antimicrobial Resistance (AMR) continues to escalate at an exponential rate now threatening to undermine the therapeutic gains made in the last few decades (Naghavi et al., 2024). Shigellosis is one of the most common causes of bacillary dysentery and represents a major public health problem worldwide, with particular challenges experienced in resource-limited settings with poor sanitation (Hendrick et al., 2025). Recent global estimates indicate that shigellosis accounts for approximately 188 million cases and over 212,000 deaths annually (Khalil et al., 2018; Kotloff et al., 2018). Two of the most common *Shigella* species include *S. flexneri* (*Shigella flexneri*) and *Shigella sonnei*, which are responsible for a large number of gastrointestinal infections (Puzari et al., 2018; Bengtsson et al., 2022). Both these species are typically spread *via* the faecal-oral route and demonstrate escalating levels of antimicrobial resistance to fluoroquinolones and other first-line agents (Ud-Din et al., 2013; Salleh et al., 2022). *Shigella flexneri* is also the dominant etiological agent in low- and middle-income countries and is associated with many outbreaks and high mortality rates amongst young children (Bengtsson et al., 2022). The rising burden of AMR in *S. flexneri*, specifically the alarming increase in fluoroquinolone resistance, has resulted in severe limitations on available treatments and complicates the management of *S. flexneri* caused infections (Divya et al., 2015; Puzari et al., 2018; Salleh et al., 2022). According to the 2024 update of the World Health Organisation (WHO)’s Bacterial Priority Pathogens List (BPPL), the WHO re-classified fluoroquinolone-resistant *Shigella* species from “medium” to “high”, indicating that they are increasingly contributing to the global AMR crisis (WHO Bacterial Priority Pathogens List, 2024). Recent surveillance data collected in 2024 from Bangladesh demonstrated that nearly all (>96%) *S. flexneri* clinical isolates were resistant to ciprofloxacin, one of the primary fluoroquinolones used to treat infections caused by *S. flexneri* (Afrad et al., 2024). Additionally, this pattern of AMR in *S. flexneri* is developing into a global issue, especially in middle-income countries. Ciprofloxacin, a widely prescribed fluoroquinolone antibiotic for the treatment of a variety of bacterial infections, induces bactericidal activity against bacteria by disrupting the action of bacterial Type II Topoisomerases, specifically DNA Gyrase and Topoisomerase IV (Hooper and Jacoby, 2016). DNA Gyrase, which consists of the GyrA and GyrB subunits in Gram-negative bacteria such as *S. flexneri*, is the primary target of Fluoroquinolones, while Topoisomerase IV, which consists of the ParC and ParE subunits, is a secondary target (Khodursky et al., 1995; Aldred et al., 2014; Johnning et al., 2015). These enzymes play key roles in DNA replication, supercoiling and chromosome segregation through a transient double-strand cleavage mechanism that is susceptible to quinolone or fluoroquinolone-based inhibition (Tamanna and Ramana, 2016; Bush et al., 2020). Fluoroquinolones have been the standard of care for many bacterial infections for many years by acting upon these critical bacterial enzymes (Hooper and Jacoby, 2016). The emergence of highly resistant *S. flexneri* strains to fluoroquinolones is now an obstacle to the successful treatment of *S. flexneri-*caused gastrointestinal infections. Chromosomal mutations in the quinolone resistance determining regions (QRDR) occur in approximately 75% of fluoroquinolone resistance cases. Numerous point mutations localised in the QRDRs of the gyrA (positions 67–106) and parC (positions 56–106) genes result in reduced affinities of fluoroquinolones for the enzymes, reducing the clinical efficacy of fluoroquinolone antibiotics (Yoshida et al., 1990; Willmott and Maxwell, 1993). Many common substitutions occur in GyrA, such as Ser83Leu (S83L) and Asp87Asn (D87N) and in ParC, such as Ser80Ile (S80I), which impede quinolone binding and lead to a high level of resistance to fluoroquinolones (Qin et al., 2017; Yu et al., 2020). These mutations often produce a high level of resistance and significantly diminish the therapeutic efficacy of current fluoroquinolones. Clinical isolation of *S. flexneri* strains bearing mutations involved in resistance, including some from the globally prevalent 2a serotype (Divya et al., 2015; Naha and Ramaiah, 2022). The resistance problem is further compounded by auxiliary mechanisms, including the acquisition of PMQR genes (e.g., qnr, aac(6′)-Ib-cr) and the increased expression of efflux pumps, which collectively contribute to the decreased intracellular concentration and diminished therapeutic effectiveness of quinolones (Yu et al., 2020). Collectively, all these findings indicate that there is an immediate need for the development of novel, effective and innovative antimicrobial agents to address the AMR issue in *S. flexneri*.

In order to provide a solution to this critical and complex therapeutic problem, the current research used a comprehensive machine-learning (ML) based framework to identify novel dual inhibitors of GyrA and ParC in *S. flexneri*, including the most common mutants responsible for resistance to ciprofloxacin or other quinolones in *S. flexneri*. The traditional method of single-target inhibition has become less effective due to the rapid evolution of mutations and the complexity of the multifactorial mechanisms involved in bacterial drug resistance. Therefore, the focus in medicinal chemistry has changed toward developing dual-function drugs that retain their antimicrobial properties regardless of the presence of QRDR mutations (Deshaies, 2025). ML has had a major impact on the discovery of new antibacterial agents. ML facilitates the rapid identification and prioritisation of compounds with antibacterial activity using learned structure-activity relationships, and it replaces the necessity for extensive experimental screening processes to determine compound activity (Rahman et al., 2022). When well-trained on MIC values and comprehensive sets of molecular descriptors, ML models are capable of identifying candidate scaffolds with potential activity against both wild-type and mutated targets (Joshi et al., 2024). Furthermore, the well-validation of ML models results in improved virtual screening hit rates, streamlined lead optimisation, and reduced attrition from downstream biological validations (Bilal et al., 2025). Collectively, using this multi-disciplinary approach, we aimed to find a potential therapeutic lead molecule as an antibacterial agent against fluoroquinolone-resistant *S. flexneri*.

## 2 Methods

### 2.1 Global resistance survey of fluoroquinolone-resistant *Shigella flexneri*

To analyse global quinolone resistance trends in *S. flexneri*, resistance-associated metadata were retrieved (up to 31 March, 2025). The data collected from the 15-year timeframe were obtained through the Centre for Genomic Pathogen Surveillance (CGPS)’s AMR watch interactive web application. The CGPS’s AMR watch (https://amr.watch/) is a web based interactive server that allows monitoring of priority bacterial pathogens defined by WHO and their associated AMR markers (David et al., 2025). A critical review of fluoroquinolone resistance due to chromosomal mutations, specifically point mutations in the QRDR of GyrA and ParC in *S. flexneri* was conducted using literature published over the last 15 years to cross-validate the fluoroquinolone resistance metadata (Ranjbar et al., 2016; Roy et al., 2020; Bengtsson et al., 2022). In addition to identifying mutational information, we were also able to determine which proteins can be targeted for further investigation.

### 2.2 Structural retrieval of target proteins

In this study, GyrA and ParC were selected as the primary and secondary targets, respectively. The crystal structure of *S*. *flexneri* GyrA is not available in the RSCB Protein Data Bank (RSCB-PDB; https://www.rcsb.org/). Consequently, we obtained the reference sequence [Accession ID: NP_708120] of *S. flexneri* 2a str. 301 from the NCBI database (https://www.ncbi.nlm.nih.gov/) (accessed on 3 April, 2025). Due to the absence of a high-quality 3D crystallographic protein structure with low resolution and without mutations, we conducted a literature search and identified a PDB structure with a resolution of 2.05 Å [PDB ID: 4CKL] of the *Escherichia coli* GyrA protein with Simocycline D8 bound. We performed a sequence-based similarity analysis to evaluate its suitability for this study. To assess structural equivalence, local (performed on 3 April 2025, using the online EMBOSS tool; https://www.ebi.ac.uk/jdispatcher/psa/emboss_water) pairwise sequence alignments were conducted between the sequence of 4CKL (representing the N-terminal domain of DNA gyrase subunit A) and the corresponding N-terminal domain of *S. flexneri* of GyrA. Upon confirmation through sequence alignment, the PDB structure was advanced for further study. Unnecessary water molecules and ligands were removed using the PyMOL molecular graphics system v3.1.6.1 software (https://www.pymol.org/). Additionally, the missing residues (Val30 to Arg32, Ala175 to Met178, Ala246 to Thr258, Glu283 to Ala291, Glu306 to Val316 and Arg426 to Asp429) in the protein structure of GyrA [4CKL] chain A, were modelled using UCSF ChimeraX v1.9 (https://www.cgl.ucsf.edu/chimerax/) to complete the protein structure (Meng et al., 2023). For DNA topoisomerase 4 subunit A (ParC) of *S*. *flexneri*, we searched the RSCB-PDB and UniProt (https://www.uniprot.org/) databases (accessed on 5 April 2025). In the absence of ParC’s 3D structure, we retrieved the AlphaFold-based structure [ID: AF-P0AFI4-F1-v4] from the AlphaFold Protein Structure Database (https://alphafold.ebi.ac.uk/) (Varadi et al., 2022). Subsequently, we proceeded with further analyses using the Topo IIA-type catalytic domain (amino acid residues position 31–494, our target domain) and removed other residues from the ParC’s AlphaFold structure using PyMOL v3.1.6.1 software.

### 2.3 Proteins preparation and validation

Following the retrieval and processing of the 3D protein structures of GyrA and ParC, these protein structures were validated through the use of PROCHECK, ERRAT (https://saves.mbi.ucla.edu/), and the ProSA-web (https://prosa.services.came.sbg.ac.at/) tool, an interactive web server for protein structure evaluation (accessed on 6 April 2025). PROCHECK evaluates the stereochemistry of the model of a protein based upon its geometric properties, such as the length of bonds, angle of bonds, and distribution of dihedral angles on a Ramachandran plot to ensure the model is accurate (Laskowski et al., 1993). ERRAT evaluates the accuracy of a protein structure by statistically evaluating its nonbonded interactions. The output of ERRAT includes a total score where a greater score is indicative of a better structure. It also includes a plot that indicates the parts of the protein with possible inaccuracies (Colovos and Yeates, 1993). ProSA-Web evaluates the quality of a model of a protein by calculating a Z-score which represents the overall quality of the model of the protein by evaluating the protein’s potential energy. The Z-score calculated by ProSA-Web was then compared to a set of native structures of proteins of varying sizes, thus allowing researchers to determine whether the model of the protein is of acceptable quality (Wiederstein and Sippl, 2007).

### 2.4 Selection and retrieval of mutants

The mutation information was previously compiled from the 15-year global metadata and literature on quinolone-resistant *S. flexneri*. Based on the most frequently observed mutations, the respective target proteins were mutated using the mutagenesis wizard tool available in PyMOL v3.1.6.1 software. The final wild-type protein structures were first loaded into PyMOL, and amino acid residues were visualised to confirm target positions. The mutagenesis wizard was then employed to introduce point mutations by selecting the desired residue positions and substituting them with the corresponding amino acids. Finally, the substitutions were applied, followed by visual inspection and structural verification prior to downstream computational analyses.

### 2.5 Functional impact of mutations on target protein sequences

Selected mutants were assessed using the Protein Variation Effect Analyser (PROVEAN) tool and cross-verified with Meta-SNP. The PROVEAN (http://provean.jcvi.org/index.php) tool predicts the biological functional impact of amino acid changes in proteins using a scoring system (Choi and Chan, 2015). A score below the default threshold of −2.5 indicates a deleterious effect, suggesting that the mutation is likely to adversely affect the protein’s function. In contrast, Meta-SNP (https://snps.biofold.org/meta-snp/), a machine-learning-based bioinformatics tool, serves as a meta-predictor of disease-causing variants. Meta-SNP is implemented using a 100-tree RandomForest WEKA library, trained on SV-2009 with 20-fold cross-validation. It predicts whether a point mutation or a single amino acid variation in a protein is disease-associated or neutral (polymorphic and non-synonymous). This tool also utilises a scoring system, where a score exceeding the default threshold of 0.5 indicates that the variant is disease-causing.

### 2.6 Protein backbone dynamics, folding free energy determination, and stability analysis of wild and mutants

The backbone dynamics of specific mutant proteins were assessed at the residue level using the DynaMine (https://bio2byte.be/b2btools/dynamine/) tool, accessible *via* the Bio2Byte web interface. Through this analysis, the backbone N-H *S* ^*2*^ order parameter values were calculated (Cilia et al., 2014). The *S* ^*2*^ values based on the experimental NMR chemical shift values provide information about the restrictions on the orientation of the atomic bond vectors with respect to the molecular reference frame. *S* ^*2*^ values exceeding 0.8 are indicative of very rigid conformations. For assessing how point mutations affect the stability of a protein, we used an *in silico* approach to analyse the potential impact of the mutations on protein stability by employing two predictive tools; the sequence-based SAAFEC-SEQ (http://compbio.clemson.edu/SAAFEC-SEQ/index.php) and the structure-based DynaMut2 (https://biosig.lab.uq.edu.au/dynamut2/). We used the SAAFEC-SEQ web server to predict the changes in folding-free energy and predict the changes in stability when one mutation occurs in a protein. SAAFEC-SEQ uses a gradient-boosting decision tree regressor to encode the physicochemical properties, sequence features, and evolutionary information to compute the change in stability free energy, or thermodynamic stability, produced by individual mutations (Li et al., 2021). We also used DynaMut2 to investigate the changes in stability and flexibility generated by mutations. This allows for analysis of flexibility analysis, and visualisation of intrachain loss or gain in the protein structure. DynaMut2 includes normal mode analysis (NMA) and graph-based signatures to investigate the influence of mutations on the stability and dynamics of proteins (Rodrigues et al., 2021).

### 2.7 Selection of ligands

Ciprofloxacin was selected as the reference scaffold analogue, hoping to identify a potential alternative lead molecule as a therapeutic against fluoroquinolone-resistant *S. flexneri*. To allow for the screening of the analogues with respect to the structural similarity to ciprofloxacin, a 3D similarity search was performed using the PubChem database (https://pubchem.ncbi.nlm.nih.gov/) (accessed on 15 April 2025). The methodology used to evaluate the properties of the analogues included their stereochemistry, alignment of structures, and adherence to pharmacophore models. In addition to these characteristics, all of the analogues were evaluated concerning their standard physiochemical properties and Lipinski rule based filtering using the following parameters to ensure they met the criteria of being drug-like; molecular weight of 230–480 Da, 1–9 rotatable bonds, 17–34 heavy atoms, hydrogen bonding donor count of ≤5, hydrogen bonding acceptor count of 2–10, TPSA between 20 and 140 Å^2^, molecular complexity of 300–900, and XLogP between −2 and 5. Compounds passing these filters were further refined through manual curation to eliminate duplicates, known toxic or unstable derivatives, metabolic byproducts of fluoroquinolone biosynthesis, and clinically used fluoroquinolone antibiotics from the first to third generations. This process resulted in a final curated set of compounds that were structurally diverse, pharmacokinetically viable, and devoid of known fluoroquinolone scaffolds, using ciprofloxacin (PubChem CID: 2764) as a reference compound. The ligands were then prepared for bioactivity classification using a machine learning-based model.

### 2.8 Machine learning (ML) based screening

#### 2.8.1 Dataset preparation

In this study, known bioactive compounds with activity against *S. flexneri* were obtained from the ChEMBL (https://www.ebi.ac.uk/chembl/) database (ID: CHEMBL614396), along with their minimum inhibitory concentrations (MIC) data (accessed on 21 April 2025). In this study, we focused on compounds that target fluoroquinolone-resistant *S. flexneri*. However, specific datasets exclusively for resistance-associated mutations in *S. flexneri* were unavailable. Therefore, we considered compounds that exhibit antimicrobial activity against the bacterial pathogen *S. flexneri*. Subsequently, duplicates were removed from the datasets. The compounds in the training dataset were then categorised into active and inactive groups based on their minimum inhibitory concentration (MIC) values. Compounds with MIC values less than or equal to 1 μg/mL were classified as active, whereas those with MIC values greater than or equal to 128 μg/mL were designated as inactive. Finally, these two filtered datasets of active and inactive compounds were used as training datasets for model construction and validation. For the test dataset, previously curated physicochemical properties and Lipinski based filtered Ciprofloxacin 3D analogous compounds were identified from the PubChem database.

#### 2.8.2 Descriptor generation and calculation

Molecular descriptors compress fundamental properties, including the structural and physicochemical attributes of small molecules, which significantly influence their biological activities. All compounds, encompassing both the training and test datasets, were processed in the 3D Standard Format (3D-SDF) using O’Babel v3.1.1 (https://github.com/openbabel/openbabel/releases/tag/openbabel-3-1-1) and subsequently uploaded to the widely utilised open-source Padel software v2.20 (http://padel.nus.edu.sg/software/padeldescriptor) for descriptor calculations (O’Boyle et al., 2011; Yap, 2011). The purpose of this resource is to standardise all data in the database by eliminating salts, identifying aromaticity, standardising nitro groups and then enabling the extraction of 1D, 2D and 3D class-based features from the molecules in the compound dataset. There are numerous types of features including topological, geometric, electronic and structural features. In addition, structural features such as the PubChem fingerprinter can be extracted. The 1D and 2D feature set (55 features or descriptor type) includes attributes such as acidic group count, AlogP, Apol, aromatic atom count, aromatic bond count, atom count, basic group count, BCUT, fragmentation complexity, H-bond acceptor and donor count, hybridisation ratio, *etc.* These 55 descriptor types have a total of 1,444 sub-features or descriptors. The 3D feature set (8 features or descriptor type) includes autocorrelation 3D, CPSA, gravitational index, RDF, *etc.* These 8 3D descriptor types have a total of 431 sub-features or descriptors. All of these biologically relevant descriptors include key molecular properties that are important for understanding how a molecule interacts with its biological target; these include polarity, aromaticity, flexibility and lipophilicity. These descriptors provide an overall chemical representation of the molecule that is required for accurate classification during ML model development.

#### 2.8.3 ML-based model prediction and accuracy evaluation

A supervised ML methodology was used to build the prediction model. The model was developed and then tested using a test dataset with WEKA v3.8.6 (Waikato Environment for Knowledge Analysis; https://weka.en.softonic.com/) which is an open source ML toolset (Ian and Eibe Frank, 2011; WEKA 3.8.6, 2022). The feature selection process was implemented using WEKA functions, and included correlation of attributes, use of information gain as a metric, and the management of missing values. The purpose of the dimensionality reduction that resulted from the feature selection process was to retain the most important features that would provide a basis for improved performance and interpretation of the model. A variety of ML models and algorithms are available to be used with WEKA *via* a simple graphical user interface. In addition to the various ML models, WEKA also provides tools for conducting common data mining operations, including regression, clustering, classification, and data visualisation, allowing for easy and complete analysis of structured datasets. Prior to developing the model, the dataset was divided into two sections - one for training the model, and the other for testing the model. In order to select the best possible models for the identification of drug candidates with or without antibacterial properties, several different predictive models were created using a combination of ML techniques. Seven ML algorithms were selected for use in creating the models, and consisted of four decision tree-based models: Decision Stump, J48 Pruned Tree, Random Forest, and Random Tree, and three rule-based models: Rep Tree, and JRIP rules and Decision Table. All seven of the models used in this research are examples of supervised ML models. As previously mentioned, supervised ML models are often used in virtual screening due to their ability to make accurate predictions and their robustness (Oliveira et al., 2023; Obaido et al., 2024). Each of the seven models was run thirty times to evaluate their performance as predictors using a thirty-fold cross-validation (CV) procedure. Thirty-fold cross-validation was used to ensure an optimal distribution across the entire molecular descriptor space. This approach minimises variance and prevents overfitting, rendering it a reliable method for model evaluation. The performance of the models was evaluated using thirteen different statistical metrics, including Correctly Classified Instances/ACC (Accuracy), Error Rate/Incorrectly Classified Instances (%), Kappa Statistic (κ), Mean Absolute Error (MAE), Root Mean Squared Error (RMSE), Matthews Correlation Coefficient (MCC), Receiver Operating Characteristic (ROC), Precision-Recall Curve Area (PRC Area), Specificity, Sensitivity (Recall), Precision (Proportion of Correctly Predicted Positive Instances), F1 Score, and Youden’s Index. The evaluation of the models was performed using a confusion matrix that was generated for each model, and all of the performance metrics were computed to verify the consistency and reliability of the results obtained from the evaluations. Accuracy for the models was determined using the formulae that have been traditionally used with the confusion matrix, as follows:

$$\text{Accuracy or ACC }\left(\%\right)=\frac{TP+TN}{\left(TP+TN+FP+FN\right)}\times 100$$

$$\text{Error rate }\left(\%\right)=\frac{FP+FN}{\left(TP+TN+FP+FN\right)}\times 100$$

$$\text{Kappa Statistic }\left(\kappa \right)=\frac{P_{o}-P_{e}}{1-P_{e}}$$

Where, $\text{Observed agreement }\left(P_{o}\right)=\left(TP+TN\right)/N$

$$\text{Expected agreement }\left(P_{e}\right)=\frac{\left(TP+FP\right)\left(TP+FN\right)+\left(FN+TN\right)\left(FP+TN\right)}{\;N^{2}}$$

$$\text{Total }\left(N\right)=TP+TN+FP+FN$$

$$\text{MCC}=\frac{\left(TP\times TN\right)-\left(FP\times FN\right)}{\;\sqrt{\left(TP+FP\right)\left(TP+FN\right)\left(TN+FP\right)\left(TN+FN\right)}}$$

$$\text{Specificity}=\frac{TN}{\left(TN+FP\right)}$$

$$\text{Recall }\left(\text{Sensitivity}\right)=\frac{TP}{\left(\;TP+FN\right)}$$

$$\text{Precision}=\frac{TP}{\left(\;TP+FP\right)}$$

$$F1\text{ Score}=2\times \frac{\left(Precision\times Recall\right)}{\left(Precision+Recall\right)}$$

$$\text{Youde}n^{\prime }s\text{ index}=\frac{TP}{\left(TP+FN\right)}+\frac{TN}{\left(TN+FP\right)}-1$$

$$\begin{array}{ll} & *\text{TP}=\text{True positives};\text{ FP}=\text{False positives};\text{ TN}=\text{True negatives};\\ & \;\text{ FN}=\text{False negatives} \end{array}$$

In order to show and validate the variety of performance comparisons, a set of graphs/plots were created using several python libraries, including pandas v2, matplotlib v3.7.0, seaborn v0.12.2, and openpyxl v3.1.2. To measure the ability of the classifiers to distinguish active from inactive molecules, the area under the curve (AUC), and the receiver operating characteristic (ROC) were calculated in addition to the AUC values of the classifiers with the use of the AUC function to assess the discriminative ability of the classifiers. A comparative ROC curve plot was created to assess the performance of the classifiers. An AUC value of 0.5 is indicative of a poor classifier, while a value greater than 0.5 represents an improved classification ability of the classifier to correctly classify true positives and true negatives, along with reduced false positives and false negatives. One of the seven trained models was chosen for further investigation based on multiple performance metrics and accuracy. The top-performing model was subsequently used to analyse our test data sets (physicochemical properties and Lipinski filtered ciprofloxacin analogues) to identify the active and inactive compounds. The identified active compounds were examined further with the use of virtual screening.

### 2.9 Virtual screening of ML screened active ligands

The ligands were subjected to pyVSvina, a python-based tool that uses AutoDock Vina (https://vina.scripps.edu/) to virtually screen against a receptor protein (Trott and Olson, 2010). A docking-based virtual screening method was employed to produce a number of possible orientations and conformations of the ligands within the binding pockets. Following this, the compounds were first subjected to an energy minimisation process using O'Babel. Then those compounds were exported into the PDBQT format for docking analysis. In order to identify the active site of GyrA, the location of Simocyclinone D8, a previously bound gyrase inhibitor, was identified using PyMOL v3.1.6.1. Additionally, for the identification of the active site of ParC, the DogSiteScorer (https://proteins.plus/help/dogsite) tool was used and the results were further validated by another web server based tool P2Rank (https://prankweb.cz/) (Volkamer et al., 2012; Jendele et al., 2019). For both GyrA and ParC the active sites located in the QRDR region of the proteins. The grid parameters were defined as follows: X = 29.408, Y = −31.489 and Z = −38.499 for GyrA and its mutant GyrA_S83L_D87N and X = −17.215, Y = 16.212 and Z = −2.475 for ParC and its mutant ParC_S80I, with dimensions of 25 × 25 × 25 Å for molecular docking. The default energy range was 4 and the exhaustiveness value was 8, with the ligands being energy minimised using the Universal Force Field (UFF). Ciprofloxacin was utilised as the control antibiotic for both GyrA and ParC proteins and their mutants. Molecular docking of the screened compounds, generated from an ML-based model, was initially performed against GyrA and its mutant GyrA_S83L_D87N. Based on the docking results, the top ten compounds with the highest average docking scores or lowest average binding energies were selected for further docking against ParC and mutant ParC_S80I. The detailed interactions of the docked complexes were visualised using Discovery Studio Visualizer (https://www.3ds.com/products/biovia/discovery-studio/visualization), an intuitive tool for protein modelling and drug design.

### 2.10 ADMET properties evaluation and biological activity prediction

The top selected compounds exhibiting the lowest average binding energies were subjected to further screening to evaluate their ADME properties, toxicity assessment and followed by biological activity prediction. To evaluate pharmacokinetic properties and toxicity assessment, we employed Deep-PK (https://biosig.lab.uq.edu.au/deeppk/), a deep learning based pharmacokinetic properties prediction tool (Myung et al., 2024). Additionally, toxicity analysis of the several toxicity parameters of the compounds that were chosen was also conducted by the ProTox 3.0 (https://tox.charite.de/protox3/) server in order to cross validate toxicity results that were provided from Deep-PK (Banerjee et al., 2024). The ProTox 3.0 server uses structure-activity relationship data that has been curated with ML algorithms to increase predictive accuracy and minimise the use of animals for testing. The server provides both LD50 values, toxicity class and specific toxicity endpoints in the form of hepatotoxicity, cardiotoxicity, carcinogenicity, mutagenicity, and cytotoxicity. Typically, the LD50 values are given as milligrams per kilogram of body weight, where the LD50 represents the lethal dose for 50% of the experimental subjects exposed to the compound. Toxicity classes were based on the Globally Harmonised System of Classification and Labelling of Chemicals (GHS). If the LD50 ranges from 0 to 300, it would be classified as Class I-Class III and indicates fatal and/or toxic effects when ingested; if the LD50 range from 300 to 2000, it would be classified as Class IV, and indicates harmful effects if ingested; if the LD50 exceeds 2000, it would indicate potentially less harmful or non-toxic if ingested. Those compounds that exhibited minimal toxic effects compared to standard drugs were then further investigated for their potential antibacterial activities and their effects on DNA synthesis or topoisomerase activity using the PASS (https://way2drug.com/PassOnline/) online tool (Filimonov et al., 2014).

### 2.11 Density functional theory (DFT) analysis

The compound which was identified from the toxicity testing has been investigated by DFT to further refine the compound’s structure and chemical properties. The DFT technique is used as a quantum mechanical approach to investigate the structural properties of molecules, and reactivity of atoms in molecules, as well as their ground state properties and to evaluate and optimise the structural stability of molecules (Baerends and Gritsenko, 1997; Naha et al., 2022). Considering the physiological ionisation state of control Ciprofloxacin and the lead molecule, the geometry optimization and DFT calculations were performed using the deprotonated form of both molecules. This 3D structure was optimised using the DFT calculation with the B3LYP hybrid exchange functional, along with the 6-311G++ (d,p) basis set, as applied in the Gaussian 16 W v1.1 package (Frisch et al., 2016). B3LYP is recognised for being highly accurate in estimating the geometric parameters of molecular structures and their chemical properties. Several studies have suggested that the use of a larger basis set such as 6-311G++, provides higher precision due to the polarisation function which improves the accuracy of calculations close to the nucleus, thus increasing overall accuracy. Visualisation of the molecule was completed using GaussView v6.1.1 (Dennington et al., 2019). From FMO energy data, several mechanical descriptors were estimated, including the HOMO (the highest occupied molecular orbital), LUMO (the lowest unoccupied molecular orbital), ionisation potential (IP), electronegativity (χ), electron affinity (EA), chemical potential (µ), chemical hardness (η), and chemical softness (σ). In addition, analysis of the molecular electrostatic potential (MEP) was performed to estimate the sites of attack for both nucleophilic and electrophilic reactions.

### 2.12 Redocking of optimised ligand

Following the geometry optimisation of the screened compound, additional docking procedures are necessary to verify its binding potential to the target protein and its mutants. Subsequently, we employed AutoDock Vina, utilising the same active site and grid parameters, to redock the DFT-optimised screened compound. The docked complexes, which exhibited multiple intermolecular interactions, were visualised using PyMOL v3.1.6.1 and Discovery Studio Visualizer v20.1.0.19295 for three-dimensional and two-dimensional representations, respectively.

### 2.13 Molecular dynamics simulation based protein-ligand interaction analysis

Molecular dynamics (MD) simulations were performed using the GROMACS 2024.2 software (https://www.gromacs.org/) suite to examine the dynamic stability and conformational properties of the target proteins and their complexes with the lead compounds (Mark et al., 2024). The protein-ligand complexes, comprising GyrA wild type, GyrA double mutant (S83L_D87N), ParC wild type, and ParC single mutant (S80I), were prepared using the CHARMM36 force field and ligand parameters from the CGenFF (https://cgenff.com/server), with ligand topologies generated through the CGenFF server (Vanommeslaeghe et al., 2010). Each of these systems was initially embedded within a dodecahedral box having a buffer zone of approximately 1.2 nm from the edge of the box, and then solvated in water, employing the TIP3P model of water molecules. Counterions (Na^+^ and Cl^−^) were then added to neutralise the charge on each of the systems. An energy minimisation procedure was performed on each of the systems; this involved the application of the steepest descent algorithm until a maximum force per atom of 10 kJ/mol was achieved (the convergence criterion); this was accomplished through 50,000 iterations of the algorithm with the Verlet cut-off scheme used to prevent non-physical artefacts during the process. Following energy minimization of the systems, each system underwent equilibration in two different phases of equilibration: an NVT phase (where the number of particles, the volume, and the temperature are constant) at 300 K for 100,000 picoseconds, and then an NPT phase (where the number of particles, the pressure, and the temperature are constant) at 1 bar for another 100,000 picoseconds. Next, each system underwent 100 nanosecond (ns) production runs to determine the structural stability and interactions of the various systems being studied. The generated trajectories from the production runs were evaluated based on a variety of parameters including the RMSD (root mean square deviation), RMSF (root mean square fluctuation), Rg (radius of gyration), SASA (solvent accessible surface area) of each of the systems (apo forms of target proteins and the complexes). Then we further evaluated the HBPs (hydrogen bonding profiles), hydrogen bonds occupancy analysis and IE (interaction energies) of each of the complexes, in order to study the effects of mutations on the conformational behavior, stability, and interaction dynamics of both the wild-type and the mutant proteins as well as each of the docking complexes studied over time (Basu et al., 2024).

### 2.14 Principal component analysis

The application of Principal Component Analysis (PCA) to MD simulations assessed the dynamic behaviour of wild-type and mutant forms of apo and ligand-bound GyrA and ParC proteins by investigating the movement and transition dynamics between metastable states. PCA identifies the dominant, correlated motions in proteins and how they relate to protein-ligand interaction and flexibility. A covariance matrix (CM) is generated through the fluctuations in position of the protein’s Cα atoms (the atoms bonded directly to carbon atoms) to reduce the amount of “noise” present and focus the analysis on the biologically relevant movements. The CM is then diagonalised, resulting in the derivation of the eigenvectors and eigenvalues which represent the directions and magnitudes of the motions, respectively. The first two principal components (PC1 and PC2) account for most of the variance in the displacement of the protein’s atoms and provide a simple representation of the key dynamics of the protein. The Free Energy Landscape (FEL) is analysed using the first two principal components as the reaction coordinates to describe the functionally important conformational space of the protein. The FEL basins are used to define the different equilibrium states and metastable conformations of the protein, and low energy areas indicate more stable conformers. The ‘gmx covar’, ‘gmx anaeig’ and ‘g_sham’ tools of GROMACS 2024.2 software packages were used to calculate the covariance matrices, 2D projections of the trajectory onto PC1 and PC2, and 2D PCA/FELs with 3D contour plots against PC1 and PC2 as representations of the Gibbs free energy. The results provided a basis to compare the global movements and stabilities of the apo-forms of the proteins to their respective ligand-bound counterparts for both wild-type and mutant forms and provided insight into the structural dynamics and functionally important transitions that occur when a ligand binds to either form of the protein (Joshi et al., 2025). To determine whether the MD simulation had converged as well as to provide an indication of its reliability, Ci (cosine content) analysis was conducted using the principal component directions identified in the PCA. The first two principal components of the projection trajectories that resulted from the MD simulations were analysed for their cosine content. The cosine content was numerically integrated using the standard formulation, using python scripts with the trapezoidal method to perform the numerical integration. If the cosine content is <0.2, then the MD simulation has adequately sampled and converged (Maisuradze et al., 2009).

### 2.15 MM/GBSA and MM/PBSA analysis for validation of docked complexes

Binding affinities and stabilities of the potential lead compound to both wild and mutant types of GyrA and ParC proteins were investigated *via* the gmx_MMPBSA tool (https://valdes-tresanco-ms.github.io/gmx_MMPBSA/dev/) for calculation of the binding free energies (Valdés-Tresanco et al., 2021). MM/GBSA (Molecular Mechanics Generalised Born Surface Area) and MM/PBSA (Molecular Mechanics Poisson-Boltzmann Surface Area) methods were used as they are molecular mechanics-based methods that can be used to calculate the thermodynamics of the protein-ligand complexes (Genheden and Ryde, 2015). These calculations will help to understand and validate the MD simulation results and finally provide a detailed energetic view of the interactions within the complex. Calculations were performed by extracting the post-simulation data for each simulation (i.e., 100 ns), and default gmx_MMPBSA settings were used to ensure consistency and reproducibility. The system was maintained at 298.15 K. For MMGBSA calculations, the solvation parameters were set with igb = 5 and dielectric constants of 1.0 (internal) and 78.5 (external), whereas for MMPBSA, ipb = 2 with dielectric constants of 1.0 and 80.0. Both methods considered implicit solvent effects to accurately model the contributions of the polar and nonpolar solvation terms. The total binding free energy (ΔG_total) was calculated as the sum of the gas-phase energy components (van der Waals and electrostatic interactions) and solvation energy contributions (polar and nonpolar). Visualisation and energy decomposition were conducted using gmx_MMPBSA_ana to assess the relative contributions of each energetic component to the overall binding process.

## 3 Results

### 3.1 Global resistance metadata report of fluoroquinolone-resistant *Shigella flexneri*

**FIGURE 1:**
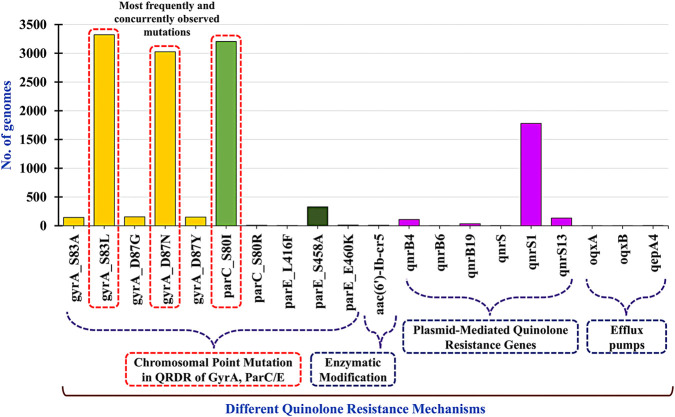
A overview of global metadata on quinolone resistance mechanisms and summarise report of *Shigella flexneri* from 2010 to 2025.

According to these metadata reports, the majority of quinolone resistance in *Shigella* spp. is attributed to chromosomal point mutations in the QRDR of GyrA/B and ParC/E, underscoring the global resistance trends (Supplementary Material S1). Among the various mechanisms contributing to quinolone resistance, approximately 72%–75% of these mutations involve S83L and D87N in GyrA and S80I in ParC. These mutations are the most prevalent and are frequently observed concurrently in quinolone-resistant *S*. *flexneri*, as illustrated in Figure 1. The double mutations S83L and D87N in GyrA and single mutation S80I in ParC were selected for further study.

### 3.2 Validation of retrieved target proteins

Alignment analyses revealed that the local pairwise alignment demonstrated 100% identity and similarity between the N-terminal domain of *E. coli* GyrA (PDB ID: 4CKL) and the corresponding region of *S. flexneri* GyrA (Supplementary Material S2 Section S1). These values significantly exceed the commonly accepted thresholds for accurate structural substitution in protein modelling, where identities above 40%–50% generally support reliable homology, and values above 90% indicate near-identical folds within the conserved regions (Abagyan and Batalov, 1997). Given the high evolutionary relatedness of *S. flexneri* and *E. coli* and the complete conservation observed across the QRDR and TOPO IIA catalytic motifs, the 4CKL structure was selected as a validated structural surrogate for *S. flexneri* GyrA. The chosen template preserves all the key catalytic residues and active site geometry required for subsequent analyses. The processed protein structure of GyrA (PDB ID: 4CKL) revealed that 85.4% and 10.1% of residues were located in the most favourable and additionally allowed regions, respectively, with 2.3% in the disallowed region of the Ramachandran plot, as evaluated using PROCHECK. For ParC (AlphaFold-based structure; ID: AF-P0AFI4-F1-v4, with very high confidence; average pLDDT score: 93.14%), 92.9% and 7.1% of residues were in the most favourable and allowed regions, respectively, with 0.0% of residues in the disallowed region (Supplementary Material S2; Supplementary Figure S1). ERRAT evaluated the overall quality factors of the GyrA and ParC proteins, yielding scores of 89.9 and 97.79, respectively, indicating the conformational accuracy and quality of the generated protein structures. The overall model quality assessment of GyrA (Experimental X-ray diffraction-based structure) and predicted protein ParC (AlphaFold-based structure) revealed Z-scores of −9.24 and −9.34 for GyrA and ParC, respectively (Supplementary Material S2; Supplementary Figure S1). This Z-score for ParC aligns closely with the range typically observed for protein structures experimentally determined using X-ray crystallography (Sippl, 1993). The local model quality, expressed in terms of stable protein conformation, was validated based on the energetics curve and topoisomerase (TOPO) IIA-type catalytic domains of GyrA and ParC, which were below the stability threshold cutoff (0.00). Once retrieved and processed for target proteins, selected mutations were then introduced into the target proteins.

### 3.3 Functional impact on target proteins due to mutation

PROVEAN has predicted that the mutations S83L and D87N in GyrA, as well as S80I in ParC, are “deleterious.” This indicates that these amino acid substitutions are likely to adversely affect protein function. Such mutations may compromise the protein’s structure, stability, or capacity to interact with other molecules, potentially resulting in altered or impaired biological activity (Ng and Henikoff, 2001; Pandurangan and Blundell, 2020). According to the Meta-SNP results, all selected mutants of GyrA and ParC were predicted to be disease causing or associated with disease. The functional impact of these mutations on the target proteins is presented in Table 1.

**TABLE 1:** Functional impact of selected mutations on the target proteins.

Webserver/Tools		PROVEAN		Meta-SNP	
Proteins	Mutations	Score (default threshold = −2.5)	Prediction	Score (default threshold = 0.5)	Prediction
GyrA	S83L	−4.203	Deleterious	0.64	Disease
	D87N	−4.544	Deleterious	0.605	Disease
ParC	S80I	−5.773	Deleterious	0.728	Disease

### 3.4 Protein backbone dynamics, folding free energy determination, and stability analysis of wild and mutants

**FIGURE 2:**
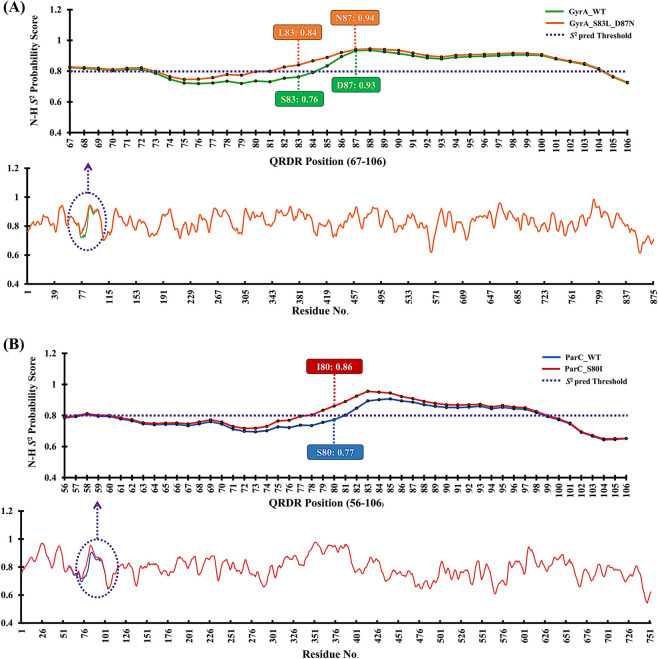
The structural flexibility of the target proteins and their mutants **(A)** relative backbone flexibility in GyrA_WT and GyrA_S83L_D87N **(B)** relative backbone flexibility in ParC_WT and ParC_S80I.

**FIGURE 3:**
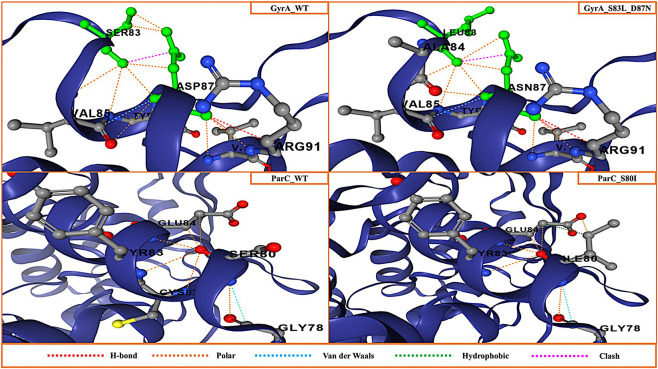
The structural impacts on target proteins resulting from the loss of intra-chain interactions due to mutations.

The relative backbone dynamics of wild-type GyrA and ParC (GyrA_WT and ParC_WT) and their mutants (MTs), specifically GyrA_S83L_D87N and ParC_S80I, exhibited no significant overall alterations in the average S^2^-probability score, which was approximately 0.829 for GyrA and 0.830 for ParC. Within the QRDR of GyrA_WT and GyrA_S83L_D87N, the S^2^-probability scores were 0.827 and 0.849, respectively. Similarly, in the QRDR of ParC_WT and ParC_S80I, the scores were 0.78 and 0.8, respectively, thereby validating the structural integrity of the proteins. However, specific mutations, such as S83L and D87N in gyrA, resulted in an increase in the S^2^-probability score from 0.762 to 0.84 and from 0.933 to 0.94, respectively. In contrast, the S80I mutation in ParC altered the score from 0.773 to 0.861 (Supplementary Material S3). The overall rigidity within the QRDR of both proteins slightly increased (Figure 2). The mutational analysis results for the single and double mutants of GyrA and ParC are summarised in Table 2. The GyrA_S83L mutation was consistently predicted to be destabilising by both SAAFEC-SEQ (ΔΔG = −0.07 kcal/mol) and DynaMut2 (ΔΔG Stability = −0.39 kcal/mol). Conflicting predictions were observed for the GyrA D87N mutation. SAAFEC-SEQ analysis predicted a destabilising effect (ΔΔG = −0.27 kcal/mol), whereas DynaMut2 suggested a minor stabilising effect (ΔΔG Stability = +0.01 kcal/mol). The differences in ΔΔG predictions for the D87N mutation in GyrA between the SAAFEC-SEQ and DynaMut2 likely arise due to fundamental methodological differences between those two tools. As SAAFEC-SEQ is a sequence-based analysis that captures physicochemical disruption from Asp to Asn (D to N) substitution, while DynaMut2 uses structural context, normal mode analysis and intramolecular interaction, suggesting that substitution in a specific residue may locally stabilise through compensatory hydrogen-bonding interactions and minimal steric disruption. The combined effect of the double mutation GyrA_S83L_D87N was evaluated. With an average distance of 5.83 Å between the mutated residues, the combined DynaMut2 analysis predicted a significant destabilizing effect, with a ΔΔG Stability of −1.34 kcal/mol, which aligns with the destabilizing prediction of the sum of the individual DynaMut2 ΔΔG values (−0.38 kcal/mol). Conversely, the ParC_S80I mutation was predicted to have a stabilizing effect by both tools, with SAAFEC-SEQ and DynaMut2 reporting ΔΔG values of +0.14 and +0.34 kcal/mol, respectively. Loss of intra-chain interactions due to mutations in GyrA and ParC was studied through the relative dynamics with the help of DynaMut2, depicted in Figure 3.

**TABLE 2:** Folding free energy determination and stability analysis of selected mutations.

Webserver/Tools		SAAFEC-SEQ		DynaMut2	
Proteins	Single mutations analysis	ΔΔG (kcal/mol)	Predicted effect	ΔΔG stability (kcal/mol)	Predicted stability change
GyrA	S83L	−0.07	Destabilizing	−0.39	Destabilizing
	D87N	−0.27	Destabilizing	0.01	Stabilizing
ParC	S80I	0.14	Stabilizing	0.34	Stabilizing

Webserver/Tools		DynaMut2			
Protein	Double mutations analysis	Average distance	Sum ΔΔG stability	Prediction ΔΔG stability	Predicted stability change
GyrA	S83L_D87N	5.83	−0.38	−1.34	Destabilizing

### 3.5 *In silico* ligand mining and pre-selection

A total of 3,688 compounds exhibiting three-dimensional similarity and structural relatedness were retrieved from the PubChem database. The ligand selection and filtering process effectively enriched the dataset for novel ciprofloxacin analogues with favourable pharmacokinetic properties. From this initial dataset of 3,688 ciprofloxacin analogues derived from PubChem, 96% (n = 3,539) successfully passed the standard physicochemical properties-based drug-likeness filters, indicating a high degree of scaffold suitability for oral therapeutic development. A final set of 3,397 analogues were identified through additional manual curation and filtering that removed all known fluoroquinolones and unwanted analogues from the initial pool, which resulted in a chemical diversity of ciprofloxacin analogues (a total of 3,397) and a new space for drug development (Supplementary Material S4). This large and diverse dataset was then used as a basis for subsequent ML based screening to identify potential active compounds against *S. flexneri* and provide an opportunity to develop better alternative treatments to existing fluoroquinolone antibiotics.

### 3.6 Evaluation of various ML classifiers’ relative performance

**FIGURE 4:**
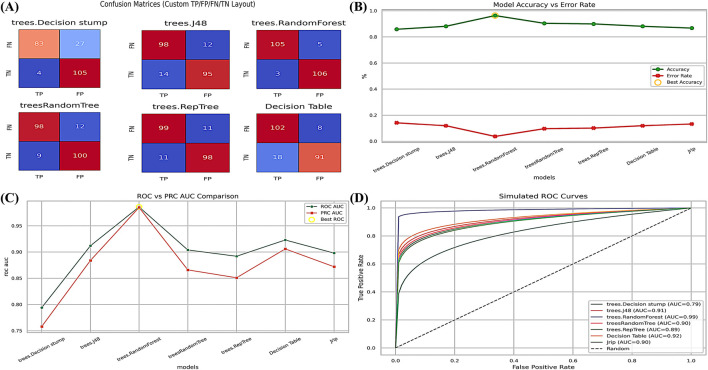
Performance comparison of all machine learning algorithms generated models **(A)** Confusion matrices of all generated models **(B)** Model accuracy vs. Error rate plot **(C)** ROC vs. PRC AUC comparison **(D)** Simulated ROC Curves.

**FIGURE 5:**
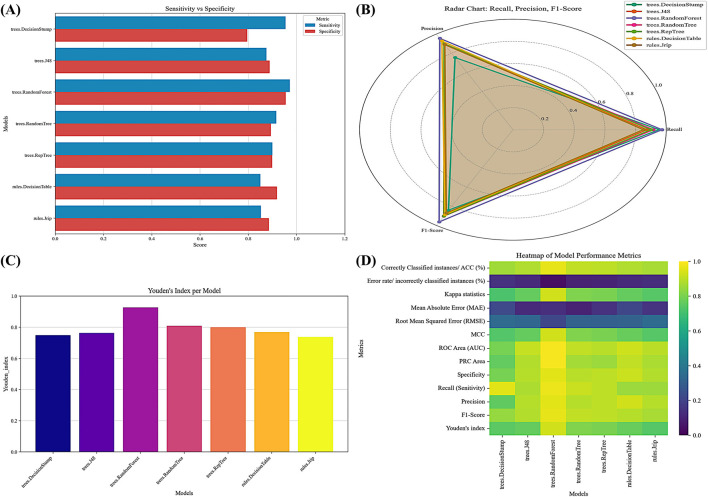
Performance comparison of all machine learning algorithms generated models **(A)** Sensitivity vs. Specificity horizontal bar chart **(B)** Radar chart of Recall, Precision and F1-Score **(C)** Bar plot of Youden’s Index per model **(D)** Heatmap of all models’ performance metrics.

The training datasets of active and inactive compounds were created by filtering 110 active and 109 inactive compounds for the development and testing of the model (Supplementary Material S5). Both datasets, along with the test set of 3,397 analogues, were used to generate descriptors (All the descriptors, including sub-features or descriptors, listed in Supplementary Material S2; Supplementary Table S1). To evaluate the different ML techniques to be used as models, the performance of each model was compared based on the training data sets and presented in Table 3. As shown by the comparison of the different models, the Random Forest technique produced the best results. The Random Forest model had the highest predictive ability at 96.35% and the lowest error rate at 3.65%, thus outperforming the other models that were tested. The Random Forest model’s kappa statistic (k = 0.9269) and Matthews Correlation Coefficient (MCC = 0.927) indicate excellent agreement between the model’s predictions and the actual classification of the compounds. The Random Forest model had the lowest root mean squared error (RMSE = 0.2137), and its Area Under the Curve (AUC = 0.986) and Precision Recall Curve (PRC = 0.985) values demonstrate that it is very accurate in predicting whether a compound is active or inactive. Other models, including Random Tree (accuracy = 90.41%, error rate = 9.59%, k = 0.8082), RepTree (accuracy = 89.95%, error rate = 10.05%, k = 0.7991), J48, and Decision Table (accuracy = 88.13%, error rate = 11.87%, k = 0.7625) performed adequately but produced greater errors than the Random Forest model and were less robust. The least complex models, JRip (accuracy = 86.76%, error rate = 13.24%, k = 0.7351) and Decision Stump (accuracy = 85.84%, error rate = 14.16%, k = 0.7172) had reduced predictability. The high specificity (S = 0.955), recall (R = 0.972), precision (P = 0.955), and F1 score (F1 = 0.963) of the Random Forest model demonstrate that it is a good generalizer and does not have a propensity for overfitting. Among all the supervised learning algorithms, Random Forest exhibited superior performance across each key metric. Performance comparison of various ML models using multiple evaluation metrics were visualised in Figures 4, 5. These findings validated the Random Forest model as the optimal predictive framework for screening ciprofloxacin analogues retrieved from the PubChem database.

**TABLE 3:** Performance comparison of various machine learning models using multiple evaluation metrics.

Models	Correctly classified instances/ACC (%)	Error rate/incorrectly classified instances (%)	Kappa statistics	Mean absolute error (MAE)	Root mean squared error (RMSE)	MCC	ROC area (AUC)	PRC area	Specificity	Recall (Sensitivity)	Precision	F1-score	Youden’s index
Decision stump	85.84%	14.16%	0.7172	0.2248	0.3417	0.733	0.794	0.758	0.795	0.954	0.755	0.842	0.749
J48	88.13%	11.87%	0.7625	0.1186	0.332	0.763	0.912	0.884	0.888	0.875	0.89	0.883	0.763
Random forest	**96.35%**	**3.65%**	**0.9269**	**0.1341**	**0.2137**	**0.927**	**0.986**	**0.985**	**0.955**	**0.972**	**0.955**	**0.963**	**0.927**
Random tree	90.41%	9.59%	0.8082	0.0959	0.3097	0.809	0.904	0.866	0.893	0.916	0.89	0.903	0.809
RepTree	89.95%	10.05%	0.7991	0.141	0.3041	0.799	0.892	0.851	0.899	0.9	0.9	0.9	0.799
Decision table	88.13%	11.87%	0.7625	0.2123	0.3095	0.766	0.923	0.906	0.919	0.85	0.927	0.887	0.769
Jrip	86.76%	13.24%	0.7351	0.1516	0.347	0.736	0.898	0.872	0.885	0.852	0.89	0.87	0.737

Based on the training data set, .the Random Forest based model produced the best performance among all predicted models.

### 3.7 ML-based screening

**FIGURE 6:**
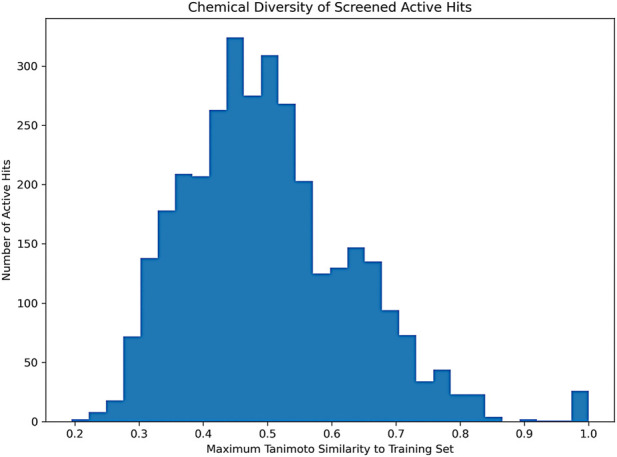
Histogram showing the structural similarity between the training set and the screened active hits to demonstrate chemical diversity.

From an initial dataset comprising 3,397 compounds, the model based on the Random Forest algorithm identified 3,336 ciprofloxacin analogues as active and 61 compounds as inactive. Consequently, the 3,336 active compounds were prioritised for subsequent virtual screening in the study (Supplementary Material S6). Figure 6 depicts the structural similarity between the training set and screened “active” hits, which demonstrates the chemical diversity.

### 3.8 Virtual screening

The molecular docking-based virtual screening data of 3,336 active compounds against GyrA and the GyrA double mutant S83L_D87N are presented in Supplementary Material S7. The ten compounds exhibiting the lowest average binding energies were selected for further docking studies against ParC and its mutant ParC_S80I, as presented in Tables 4–6.

**TABLE 4:** Top 10 compounds and control against GyrA wild and mutant S83L_D87N with avg. dock score from vHTS.

Control and ligands	GyrA_WT	GyrA_S83L_D87N	Avg. Binding affinity (kcal/mol)
Ciprofloxacin	−6.3	−6.2	−6.25
CA_1432	−7.8	−7.8	−7.8
CA_2908	−7.6	−7.7	−7.65
CA_2491	−7.5	−7.5	−7.5
CA_2177	−7.5	−7.5	−7.5
CA_2476	−7.5	−7.5	−7.5
CA_83	−7.5	−7.5	−7.5
CA_1617	−7.4	−7.5	−7.45
CA_581	−7.4	−7.5	−7.45
CA_2410	−7.4	−7.4	−7.4
CA_3218	−7.4	−7.4	−7.4

**TABLE 5:** Selected top 10 compounds against GyrA_WT and GyrA_S83L_D87N tested against ParC wild and ParC_S80I.

Control and ligands	ParC_WT	ParC_S80I	Avg. binding affinity (kcal/mol)
Ciprofloxacin	−6.1	−6.1	−6.1
CA_1432	−6.8	−6.8	−6.8
CA_2908	−6.4	−6.3	−6.35
CA_1617	−7.1	−7.1	−7.1
CA_2491	−6.6	−6.6	−6.6
CA_2177	−6.6	−6.7	−7.65
CA_2476	−7.3	−7.3	−7.3
CA_83	−6.6	−6.6	−6.6
CA_2410	−6.5	−6.5	−6.5
CA_3218	−6.2	−6.2	−6.2
CA_581	−6.6	−6.5	−6.55

**TABLE 6:** Average dock score of selected top 10 compounds against GyrA, ParC wild and their mutants.

Control and ligands	Avg. Binding affinity against GyrA WT and GyrA_S83L_D87N (kcal/mol)	Avg. Binding affinity against ParC WT and ParC_S80I (kcal/mol)	Avg. Binding affinity against GyrA, ParC and their mutants (kcal/mol)
Ciprofloxacin	−6.25	−6.1	−6.18
CA_1432	−7.8	−6.8	−7.3
CA_2177	−7.5	−7.65	−7.58
CA_581	−7.45	−6.55	−7
CA_2491	−7.5	−6.6	−7.05
CA_1617	−7.45	−7.1	−7.28
CA_2476	−7.5	−7.3	−7.4
CA_2410	−7.4	−6.5	−6.95
CA_83	−7.5	−6.6	−7.05
CA_3218	−7.4	−6.2	−6.8
CA_2908	−7.65	−6.35	−7

### 3.9 ADMET evaluation and biological activity prediction

The selected top ten compounds’ pharmacokinetic properties and toxicity assessment results were depicted through Deep-PK, presented in Supplementary Material S2; Supplementary Table S2. Among those ten compounds, only one compound, CA_1617 (Ciprofloxacin Analogue 1617, PubChem CID: 13500666), satisfied the established toxicity parameters when further cross-validated with ProTox 3.0. This compound exhibited a toxicity value of 4,000 mg/kg, classified as class V, with no evidence of carcinogenicity or mutagenicity. The toxicity assessment included evaluations of hepatotoxicity, cardiotoxicity, carcinogenicity, mutagenicity, and cytotoxicity. A comprehensive compilation of the biological activity assessment and toxicity prediction (through ProTox 3.0) for the top ten selected compounds, along with the control antibiotic, is presented in Table 7. All top ten compounds demonstrated promising antibacterial properties, specifically through the inhibition of topoisomerase II, as predicted by *in silico* analysis.

**TABLE 7:** Biological activity prediction and toxicity assessment.

Compound code	PubChem IDs	Avg dock score	Biological activity Pa>0,3	Predicted LD50	Predicted tox class	Hepato-toxicity	Cardio-toxicity	Carcino-genicity	Muta-genicity	Cyto-toxicity
Ciprofloxacin	2,764	−6.18	Proven	2000	4	Inactive	Inactive	Inactive	Active	Inactive
CA_1432	11796525	−7.3	Antibacterial, topoisomerase II inhibitor, DNA synthesis inhibitor, antimycobacterial	4,336	4	Inactive	Inactive	Inactive	Active	Inactive
CA_2177	18329314	−7.58	Antibacterial, topoisomerase II inhibitor, DNA synthesis inhibitor	700	4	Active	Inactive	Inactive	Inactive	Inactive
CA_581	502070	−7	Antibacterial, topoisomerase II inhibitor, DNA synthesis inhibitor, antimycobacterial, DNA gyrase inhibitor, antituberculosic	2000	4	Inactive	Inactive	Inactive	Active	Inactive
CA_2491	44371371	−7.05	Antibacterial, topoisomerase II inhibitor, DNA synthesis inhibitor, antimycobacterial	4,000	5	Inactive	Inactive	Inactive	Active	Inactive
CA_2476	44367335	−7.4	Antibacterial, topoisomerase II inhibitor, DNA synthesis inhibitor, antimycobacterial, antibiotic quinolone-like, DNA gyrase inhibitor, antituberculosic, cell wall synthesis inhibitor, DNA topoisomerase IV inhibitor, bacterial efflux pump inhibitor, topoisomerase I inhibitor	1,000	4	Inactive	Inactive	Inactive	Inactive	Inactive
**CA_1617**	**13500666**	**−7.28**	**Antibacterial, topoisomerase II inhibitor, DNA synthesis inhibitor, antimycobacterial, bacterial efflux inhibitor**	**4,000**	**5**	**Inactive**	**Inactive**	**Inactive**	**Inactive**	**Inactive**
CA_2410	44338540	−6.95	Antibacterial, topoisomerase II inhibitor, DNA synthesis inhibitor, antimycobacterial, antibiotic quinolone-like, DNA gyrase inhibitor, antituberculosic	3,500	5	Inactive	Inactive	Inactive	Active	Inactive
CA_83	384539	−7.05	Antibacterial, topoisomerase II inhibitor, DNA synthesis inhibitor, antimycobacterial, antituberculosic	1,556	4	Inactive	Inactive	Inactive	Active	Inactive
CA_3218	145976317	−6.8	Antibacterial, topoisomerase II inhibitor, DNA synthesis inhibitor, antimycobacterial	4,000	5	Active	Inactive	Inactive	Active	Inactive
CA_2908	1,01619401	−7	Antibacterial, topoisomerase II inhibitor, DNA synthesis inhibitor, antimycobacterial, antibiotic quinolone-like, DNA gyrase inhibitor, antituberculosic	1,414	4	Inactive	Inactive	Inactive	Active	Inactive

CA_1617, satisfied the established toxicity parameters (showed inactive in all major toxicity assessment) among the top ten selected compounds.

### 3.10 Ligand optimisation upon DFT simulation

**FIGURE 7:**
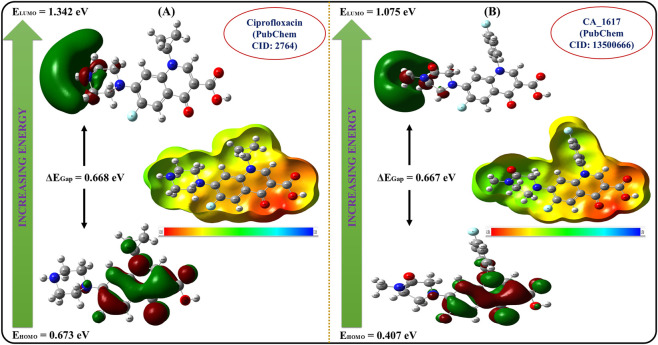
Quantum simulations highlighting the energy gap based on the frontier molecular orbital (HOMO-LUMO) and electron density map of **(A)** Control antibiotic Ciprofloxacin **(B)** Lead molecule CA_1617.

The lead compound, CA_1617 with control Ciprofloxacin were analysed using DFT for the molecular structure optimisation and the investigation of the compound’s properties including electronic structure, chemical reactivity, and the molecular interaction properties. The application of Frontier Molecular Orbitals (FMO) provided information about the ability of the molecule to donate electrons (HOMO) and accept electrons (LUMO). The HOMO-LUMO gap energy is indicative of the interactions that are stabilised when forming a protein-ligand complex. Lower HOMO-LUMO gap energies indicate higher reactivity of the molecule and lower stabilisation of the ligand-protein interaction (Perepichka and Bryce, 2005; Mohamed et al., 2025). Similar to the energy gap of the positive control antibiotic ciprofloxacin (0.668 eV), the lead compound CA_1617 had an energy gap (0.667 eV) similar to ciprofloxacin demonstrating similar electronic stability profiles. However, as the lead molecule CA_1617s energy gap is slightly higher than that of the control ciprofloxacin, indicating that the lead molecule could be more reactive than the control. Additionally, CA_1617 demonstrated a low chemical potential, therefore it supports the effectiveness of the CA_1617 in interacting with proteins. Molecular Electrostatic Potentials (MEPs) were utilised to graphically depict the relative polarity of the molecules through correlation of their electronegativity, dipole moment, and molecular charge distribution (Figure 7). Table 8 presents the global reactivity parameters for the control antibiotic and the most promising lead compound, CA_1617.

**TABLE 8:** DFT Analysis and comparison of control and lead molecule.

Compound code	HOMO*	LUMO*	ΔE*	Ionisation potential (I)*	Electron affinity (A)*	Electro-negativity (χ)*	Chemical potential (μ)*	Global hardness (η)*	Softness (Ѕ)*	Electro-philicity (ω)*
Ciprofloxacin	0.673	1.342	0.668	−0.673	−1.342	−1.007	1.007	0.334	2.993	1.518
CA_1617	0.407	1.075	0.667	−0.407	−1.075	−0.741	0.741	0.334	2.995	0.822

* All units are reported in eV.

### 3.11 Binding profiles and interactions of redocked complexes

**FIGURE 8:**
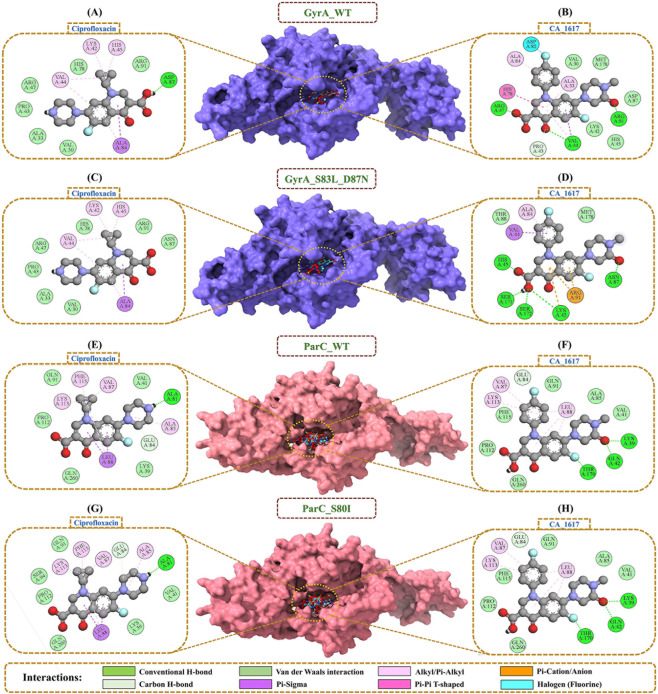
Molecular docking interaction profiles (2D and 3D) of all the complexes **(A)** GyrA_WT-Ciprofloxacin **(B)** GyrA_WT-CA_1617 **(C)** GyrA_S83L_D87N-Ciprofloxacin **(D)** GyrA_S83L_D87N-CA_1617 **(E)** ParC_WT-Ciprofloxacin **(F)** ParC_WT-CA_1617 **(G)** ParC_S80I-Ciprofloxacin **(H)** ParC_S80I-CA_1617.

Redocking was conducted to verify and confirm the potential of CA_1617 to inhibit GyrA and ParC, and their mutants, as indicated during virtual screening. The docking results, summarised in Table 9, revealed distinct differences in the interaction strength and residue engagement between the control antibiotic ciprofloxacin and the lead compound CA_1617. Across all complexes, CA_1617 exhibited stronger binding affinities (ranging from −7.1 to −7.4 kcal/mol) than ciprofloxacin (−6.2 to −6.3 kcal/mol), suggesting enhanced ligand–protein stabilisation. The intermolecular interactions of the redocked control ciprofloxacin and the CA_1617 molecule with the respective proteins and their mutants are depicted in Figure 8 in both 2D and 3D formats. For GyrA wild-type, ciprofloxacin displayed a binding energy of −6.3 kcal/mol, forming a key hydrogen bond with Asp87, a residue known to interact with quinolones.

**TABLE 9:** Redocking and interactions.

Control and lead compound	Target proteins (wild and mutant)	Binding affinity (kcal/mol)	Conventional/carbon H-bond	Van der Waals interactions	Other interactions
Ciprofloxacin (Control)	GyrA_Wild	−6.3	Asp87	Val30, Ala33, Pro43, Arg47, His78, Arg91	Pi-sigma: Ala84 Alkyl or Pi-Alkyl: Lys42, His45
	GyrA_S83L_D87N	−6.2	—	Val30, Ala33, Pro43, Arg47, His78, Asn87, Arg91	Pi-sigma: Ala84 Alkyl or Pi-alkyl: Lys42, Val44, His45
	ParC_Wild	−6.2	Ala81, Glu84	Lys39, Val41, Gln91, Pro112, Gln260	Pi-sigma: Leu88 Alkyl or Pi-Alkyl: Ala85, Val87, Lys113, Phe115
	ParC_S80I	−6.2	Ala81, Glu84	Lys39, Val41, Gln91, Ser94, Pro112, Gln260	Pi-sigma: Leu88 Alkyl or Pi-Alkyl: Ala85, Val87, Lys113, Phe115
CA_1617 (lead Compound)	GyrA_Wild	−7.3	Pro43, Val44, Arg47, Arg91	Val30, Lys42, His45, Asp87, Met178	Pi-Pi T-shaped: His78 Pi-alkyl: Ala33, Ala84
	GyrA_S83L_D87N	−7.4	Lys42, His45, Asn87, Ser171, Ser171	Thr88, Met178	Pi-cation: Arg91 Pi-sigma: Val44 Pi-alkyl: Ala84
	ParC_Wild	−7.1	Lys39, Thr170, Gln260	Ala85, Ser94, Phe115	Pi-sigma: Leu88 Alkyl or Pi-alkyl: Pro112, Lys113 Pi-Anion: Glu84
	ParC_S80I	−7.1	Lys39, Thr170, Gln260	Val41, Ala85, Ser94, Phe115	Pi-sigma: Leu88 Alkyl or Pi-alkyl: Pro112, Lys113 Pi-anion: Glu84

Additionally, several further interactions were mediated by van der Waals interactions with other residues including Val30, Ala33, Pro43, Arg47, His78 and Arg91, and also π-σ and π-alkyl interactions with Ala84, Lys42 and His45. The double mutant (S83L_D87N) was found to have an identical interaction profile to that of the original protein but it did show a clear reduction in stability due to the loss of direct hydrogen bonding in the catalytic pocket region. Conversely, CA_1617 was shown to bind much more tightly to both wild type and mutant GyrA protein (−7.3 and −7.4 kcal/mol, respectively). A number of hydrogen bonds were formed by CA_1617 to the wild type protein at positions Pro43, Val44, Arg47 and Arg91 in addition to hydrophobic and π-π stacking interactions to His78, Ala33 and Ala84. Overall, this indicates that CA_1617 has a more precise and therefore more stable fit than S83L_D87N within the active site region. Additionally, although the mutant GyrA_S83L_D87N had significantly reduced affinity for most ligands, CA_1617 showed no significant loss of affinity in its ability to form hydrogen bonds with Lys42, His45, Asn87 and Ser171 in addition to a stabilising π-cation interaction with Arg91 indicating adaptability in spite of resistance associated mutations.

These interacting residues were also similar to those previously identified for the gyrase inhibitor Simocyclinone D8, which included Lys42, His45, His80, Asn169, and Ser179 (forming H-bonds), Pro79, Gly81, Asp82, Thr88, Met92, Ser171, and Tyr266 (van der Waals interactions), Arg91 (π-cation), Gly170 (amide-π stacked), and Val44, His78, Ala84, and Leu98 (alkyl or π-alkyl interactions), as depicted in Supplementary Material S2; Supplementary Figure S2. Although the control compound (ciprofloxacin) showed moderate binding affinity (−6.2 kcal/mol) for the wild type ParC protein as well as the S80I mutant ParC protein, the lead compound (CA_1617) demonstrated significantly better binding affinity (−7.1 kcal/mol) than the control for both the wild type ParC protein and the S80I mutant ParC protein. Hydrogen bonding between CA_1617 and Lys39, Thr170, and Gln260 was also observed; in addition, hydrophobic contact was seen between CA_1617 and Ala85, Ser94 and Phe115. The additional interactions with Leu88 and Glu84 *via* π–σ and π–anion interactions likely contributed to the increased stability of CA_1617 at the catalytic site of ParC. Most importantly, it is clear that no disruption to the critical contacts between CA_1617 and the ParC protein occurred upon introduction of the S80I mutation, suggesting a degree of tolerance of this mutation and therefore preserved ability to effectively bind to the protein. Therefore, the docking results clearly support continued research into the potential dynamics and stabilities of CA_1617 to be assessed by molecular dynamics (MD) simulations.

### 3.12 Structural stability assessment through MD simulations

**FIGURE 9:**
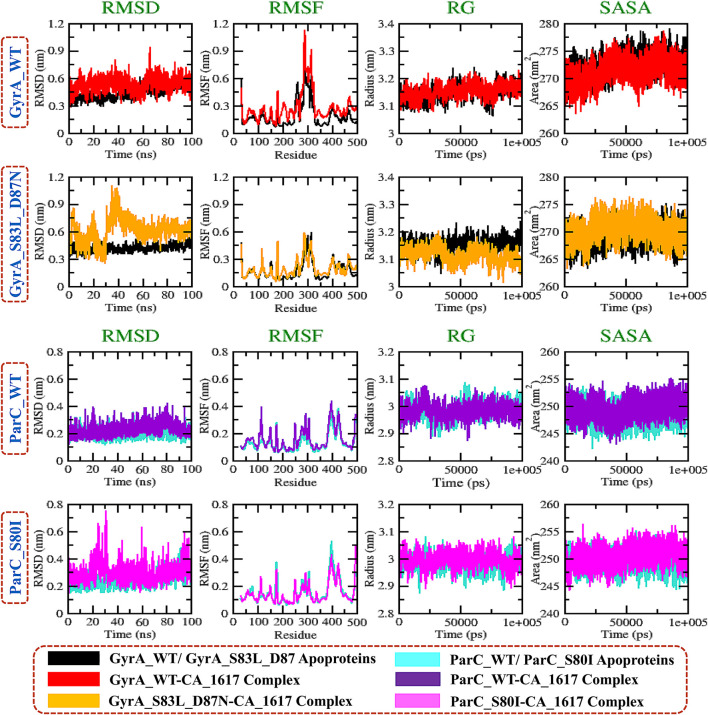
Molecular dynamics simulation profiles of all the apo forms and complexes of GyrA, ParC and their mutants with lead molecule CA_1617.

**FIGURE 10:**
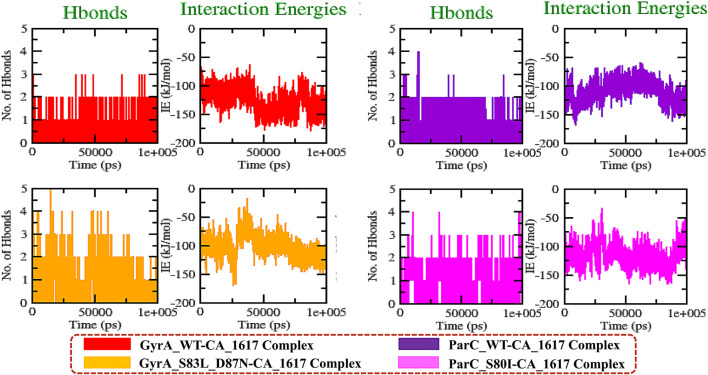
Intermolecular interactions (H-bonds) and interaction energy profiles of all the complexes.

The MD simulation provided substantial insight into the dynamic stability and flexibility, as well as the interaction profiles of both wild-type and mutant versions of GyrA and ParC proteins in combination with the lead compound CA_1617 shown in Figures 9, 10; in addition, the interaction stability parameters for each of the protein complexes formed with CA_1617 were provided in Supplementary Material S2; Supplementary Table S3. Comparing the apo-protein and protein-ligand complexes allowed for an understanding of the structural and energetic effects of ligand binding and mutations. The RMSD values indicated that all of the simulated systems achieved a stable equilibrium within the 100 ns time frame indicating good convergence of the simulations. For the wild-type GyrA, the average RMSD increased from 0.44 nm (apo) to 0.54 nm (complexed with CA_1617), representing small conformational changes after the ligand binds to GyrA. Similarly, for the double mutant GyrA_S83L_D87N, the RMSD increased from 0.418 nm (apo) to 0.612 nm (complexed with CA_1617) demonstrating greater flexibility and possible structural rearrangements due to the presence of the two mutations. Conversely, the apo wild-type and mutant ParC, along with the complexes formed with the ParC and CA_1617 had very low RMSD values indicating the proteins have high structural stability. The ParC wild-type complex demonstrated a slight increase in RMSD from 0.197 nm (apo) to 0.227 nm (complexed with CA_1617); similarly, the ParC_S80I mutant demonstrated a slight increase from 0.237 nm (apo) to 0.302 nm (complexed with CA_1617) (Figure 9). The limited fluctuations indicate that the binding of CA_1617 did not greatly alter the backbone conformation of ParC. The RMSF analysis demonstrated moderate levels of flexibility at the individual amino acid level for all of the apo forms and complexes examined. For the wild-type GyrA, the apo and complex forms demonstrated RMSF values of between 0.185 nm and 0.272 nm; for the GyrA_S83L_D87N apo and complex forms, the RMSF values were between 0.167 nm and 0.191 nm. In contrast, the wild-type apo and complex ParC demonstrated slightly lower fluctuations (RMSF = 0.133–0.153 nm); the mutant apo and ParC complexed with CA_1617 demonstrated RMSF values of between 0.152 nm and 0.146 nm (Figure 9). These data demonstrate that ParC is a more rigid molecule than GyrA. The binding of the ligand slightly increased the flexibility of the loop regions, which may be related to local conformational adjustments occurring in the binding pocket region of the proteins. The Rg values remained almost identical over the entire trajectory for all of the complexes examined and are indicative of the fact that the proteins maintained their compactness and overall tertiary structure after ligand binding. The Rg values determined for the wild-type GyrA apo and complex ranged from 3.148 nm to 3.154 nm; the Rg values determined for the double mutant GyrA_S83L_D87N apo and complex ranged from 3.153 nm to 3.113 nm; these results demonstrate only minor deviations. In contrast, the Rg values determined for the wild-type ParC apo and complex were between 2.987 nm and 2.983 nm; the Rg values determined for the mutant ParC_S80I apo and complex ranged from 2.984 nm to 2.997 nm; again, these results demonstrate only minor deviations in the global fold over the course of the simulation (Figure 9). The compactness analysis inferred from the Rg profile was also supported by the SASA trajectories, which demonstrated the formation of a uniform solvation shell surrounding all of the complexes examined. Additionally, the SASA analysis supported the RMSF results described above. The SASA values for the wild-type GyrA apo and complex were approximately 272.3 nm^2^–271.3 nm^2^; the SASA values for the double mutant GyrA_S83L_D87N apo and complex were approximately 269.22 nm^2^–270.26 nm^2^. The SASA values for the wild-type ParC apo and complex were between 248.35 nm^2^ and 249.55 nm^2^; the SASA values for the mutant ParC_S80I apo and complex were slightly higher, ranging from 249.56 nm^2^ to 250.49 nm^2^. Minor reductions or no increases in SASA values were observed in all of the ligand bound complexes relative to their respective apo forms for both GyrA and ParC, which suggest a compact arrangement and decreased solvent accessibility when forming the complexes (Figure 9). The consistent hydrogen bonding found among all of the docked complexes examined played a critical role in establishing the binding affinity between CA_1617 and the GyrA and ParC proteins, as well as their respective mutant variants. The studies showed that the hydrogen bond interactions between CA_1617 and the GyrA_WT-CA_1617 and GyrA_S83L_D87N-CA_1617 were consistent for the entire duration of the simulations; i.e., GyrA_WT-CA_1617 exhibited 2 consistent H-bonds out of a max of 3 and GyrA_S83L_D87N-CA_1617 exhibited 5 consistent H-bonds out of a max of 5; similarly, the H-bond interactions between ParC_WT-CA_1617 and ParC_S80I-CA_1617 exhibited 2 consistent H-bonds out of a max of 4. These results demonstrate the ability of these H-bond interactions to persist over time, and as such, provide evidence of their role in maintaining the structural integrity and specificity of the complexes formed with CA_1617 (Figure 10). To evaluate the persistence of the identified hydrogen bonds, occupancy analysis was performed throughout the 100 ns simulation trajectory. The lead molecule CA_1617 maintained highly stable interactions (occupancy >40%) with key residues, specifically Val44 in both wild-type (43.46%) and mutant GyrA (48.25%), and Lys39 in wild-type (41.36%) and mutant ParC (60.54%). These high-occupancy bonds serve as the primary structural anchors, ensuring the stability of the inhibitory complex even in the presence of QRDR mutations. These findings indicate that ligand stability is driven by persistent key hydrogen bonds alongside complementary noncovalent interactions. Hydrogen bond occupancy analysis results presented in Supplementary Material S8.

Analysis of the interaction energy (IE) was used to determine the relative strengths of the complexes formed. The GyrA_WT-CA_1617 exhibited the lowest IE of −126.7 kJ/mol; the GyrA_S83L_D87N-CA_1617 exhibited an increase in IE to −103.51 kJ/mol; the ParC_WT-CA_1617 exhibited an IE of −111.08 kJ/mol; and the ParC_S80I-CA_1617 exhibited the highest IE of −117.26 kJ/mol (Figure 10) (Supplementary Material S2; Supplementary Table S3).

### 3.13 Metastable conformational behaviours assessment

**FIGURE 11:**
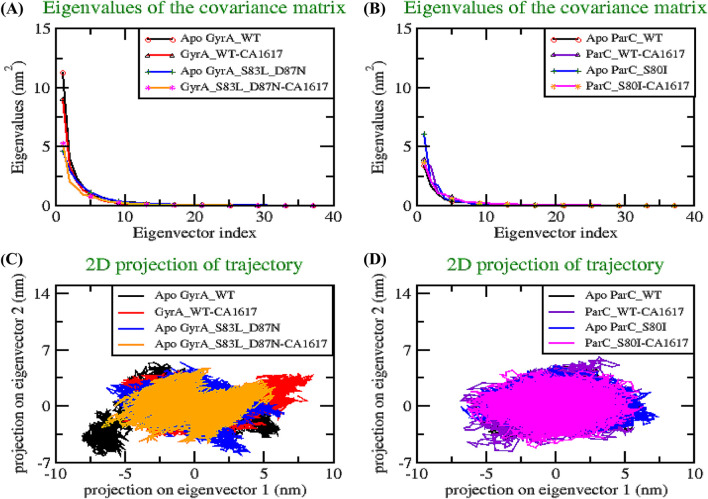
Metastable conformational behaviours assessment of all the apo forms of wild and mutant target proteins and complexes with lead molecule CA_1617 **(A)** Eigenvalues plot of apo and complexes of GyrA_WT and GyrA_S83L_D87N **(B)** Eigenvalues plot of apo and complexes of ParC_WT and ParC_S80I **(C)** 2D projection of protein motion phase of apo and complexes of GyrA_WT and GyrA_S83L_D87N **(D)** 2D projection of protein motion phase of apo and complexes of ParC_WT and ParC_S80I.

**FIGURE 12:**
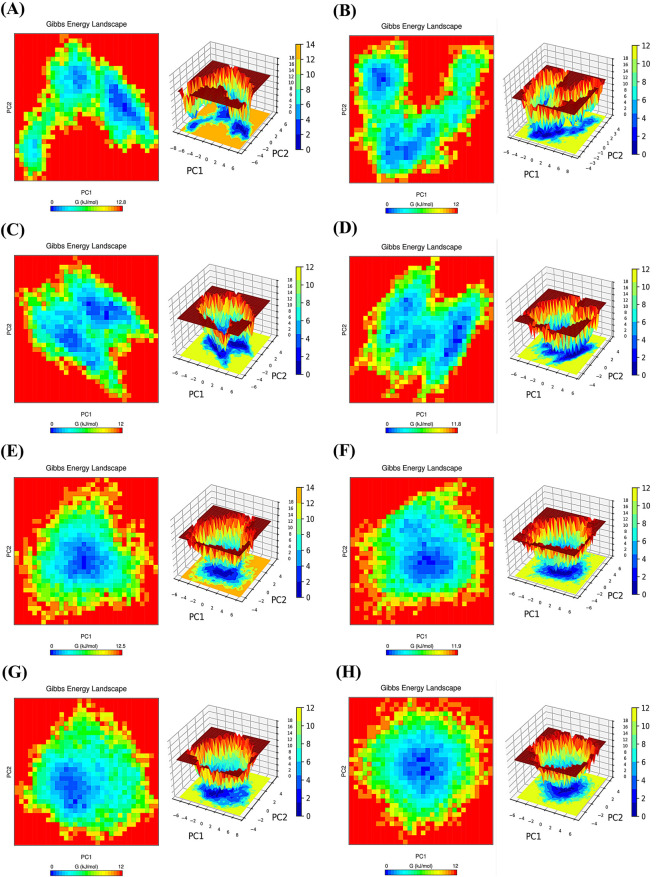
PCA - FEL contour plots (2D and 3D) of **(A)** Apoprotein GyrA_WT **(B)** GyrA_WT-CA_1617 complex **(C)** Apoprotein GyrA_S83L_D87N **(D)** GyrA_S83L_D87N -CA_1617 complex **(E)** Apoprotein ParC_WT **(F)** ParC_WT-CA_1617 complex **(G)** Apoprotein ParC_S80I **(H)** ParC_S80I-CA_1617 complex.

Distinctive conformational behaviour between the apo-protein and the CA_1617-bound complexes of GyrA and ParC in wild-type and mutant forms were discernible through the PCA projections. Each set of trajectories projected onto the first two principal components (PC1 and PC2) presented well-defined clusters, which suggested dominant motion patterns for each system. The Eigenvalue distribution and phase-space projections provided a quantitative representation of the extent and magnitude of atomic displacement, which was sampled during the course of the MD trajectory. As evident from the Eigenvalue plot, there was a sharp drop-off in value for the remaining components, indicating that the majority of the total motion could be explained by the first two principal components (Figure 11). It is this observation that supports the notion that the major dynamic motions of each protein are adequately described in a low-dimensional subspace, thereby supporting the convergence and physical validity of the simulations. The apo-forms of both GyrA and ParC had higher Eigenvalue values than their ligand-bound complexes, suggesting greater and more extensive degrees of conformational flexibility in the absence of the ligand. Conversely, the protein-CA_1617 complexes displayed a significantly sharper decay in Eigenvalues, suggesting greater constraint in terms of internal motion and greater structural compaction as a result of ligand binding. The projection of the trajectories onto the first two principal components (PC1 and PC2) clearly illustrated significant differences in the conformational sampling of the apo and bound states. The apo-forms of GyrA and ParC explored a much larger and more scattered conformational space, which indicated multiple accessible conformational states and greater dynamic heterogeneity (Figure 11). On the other hand, the CA_1617-bound complexes explored a much smaller and more confined area of conformational space, which indicated less flexibility and a greater stabilisation of the protein backbone due to ligand-induced rigidity. The mutant variant GyrA_S83L_D87N had a more compact conformation than the wild-type, while the ParC exhibited a slightly larger distribution than the wild-type. The mutations caused some structural plasticity, but ligand binding to these mutants resulted in a significant narrowing of the conformational spread, which indicated the restoration of dynamic stability. Additional analysis of the thermodynamic stability and conformational preference of the proteins was performed using FEL plots that were created as a function of the first two principal components (PC1 and PC2) (Figure 12). The resulting energy landscapes showed distinct basins that corresponded to the metastable conformational states. The apo-forms of both GyrA and ParC showed multiple shallow and more diffused energy landscapes with multiple local minima, which suggested a large degree of conformational flexibility and the existence of many energetically equivalent microstates at the active site. This observation implies that, in the absence of a ligand, these proteins may frequently switch between different structural configurations. Upon binding to the lead inhibitor molecule CA_1617, the energy surfaces became more compact, with fewer and deeper energy minima. This transition is indicative of a shift toward more energetically favourable and stable conformations, which is consistent with ligand-induced stabilisation. Of the four complexes examined, CA_1617-GyrA_Wild and CA_1617-ParC_Wild showed the most defined basins with the lowest Gibbs free energy values, which indicated the greatest stabilisation effects upon ligand binding.

Although the energy landscapes of the mutant complexes (Figures 12D,H) were slightly more rugged than those of their corresponding apo-forms (Figures 12C,G). They all showed narrower energy wells than their corresponding apo-forms, which confirmed partial recovery of stability, despite the structural changes induced by the mutations.

GyrA_S83L_D87N mutant complex (Figure 12D) showed a wider, but shallower energy basin than its wild-type counterpart (Figure 12B), which indicated that the mutations somewhat weakened the conformational constraint imposed by CA_1617. Conversely, the ParC_S80I mutant complex (Figure 12H) showed an energy profile comparable to that of the wild-type counterpart (Figure 12F), which indicated that this single mutation had little impact on the overall structural stabilisation of the complex upon ligand binding. The ability of the lead inhibitor molecule CA_1617 to restore a deep, stable energy minimum in the mutant proteins indicates that its bridge-independent binding mode provides enough compensatory energy to overcome the structural perturbations caused by mutations such as S83L or D87N for GyrA and S80I for ParC. This showcases the lead molecule’s potency against resistant *Shigella* strains to maintain a stable inhibitory complex.

The calculated cosine content values demonstrated that the majority of the protein-ligand complexes exhibited values below 0.2, except the ParC_Wild-CA_1617 complex, which was slightly higher than 0.2 (less than 0.4), confirming adequate sampling of conformational space and convergence of the MD trajectories. The cosine content values were presented in Table 10.

**TABLE 10:** Cosine content analysis of PCA projections to assess MD convergence across simulated systems.

Apo-proteins/Protein-ligand complexes	Cosine content (PC1)	Cosine content (PC2)	Convergence
Apo GyrA_WT	0.0017	0.0338	Converged
GyrA_WT-CA_1617	0.0226	0.1328	Converged
Apo GyrA_S83L_D87N	0.0772	0.2786	Moderately converged
GyrA_S83L_D87N-CA_1617	0.0003	0.1440	Converged
Apo ParC_WT	0.1514	0.0184	Converged
ParC_WT-CA_1617	0.2102	0.2866	Moderately converged
Apo ParC_S80I	0.2757	0.0279	Moderately converged
ParC_S80I-CA_1617	0.0402	0.1014	Converged

### 3.14 Binding free energy calculation

**FIGURE 13:**
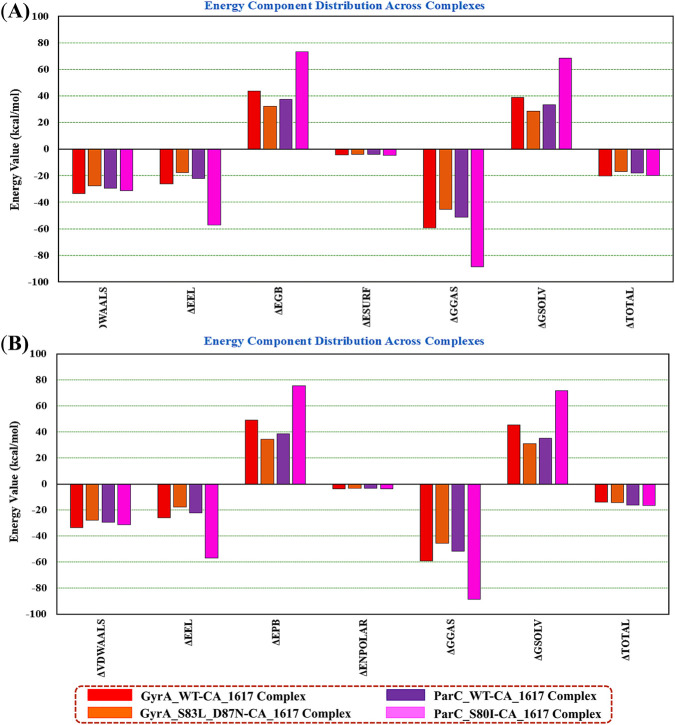
Binding free energy assessment of all the target proteins and their mutant complexes with the lead molecule CA_1617 **(A)** Based on the MM/GBSA method **(B)** Based on the MM/PBSA method.

Binding free energy calculations using MM/GBSA and MM/PBSA techniques provided complementary information about the thermodynamic stabilities of complexes between CA_1617 and the GyrA and ParC proteins, as well as their respective mutant forms (Figure 13). Both methodologies resulted in negative total binding free energies for each of the complexes studied, indicating that CA_1617 bound spontaneously and at an energetically favorable manner to each of the protein-ligand complexes listed in Tables 11, 12. Total binding free energies derived from the MM/GBSA methodology indicated that the van der Waals (ΔVDWAALS) and electrostatic (ΔEEL) components contributed significantly to the overall binding stability; however, this was largely counteracted by the solvation energy terms (ΔEGB and ΔESURF). The GyrA_Wild-CA_1617 complex had the most favourable total binding free energy (−20.36 ± 4.87 kcal/mol), followed closely by the GyrA_S83L_D87N mutant complex (−16.81 ± 4.25 kcal/mol). The ParC_Wild-CA_1617 complex also had strong binding with a free energy of −18.06 ± 3.17 kcal/mol, whereas the ParC_S80I-CA_1617 complex exhibited a moderate increase in total binding free energy (−20.02 ± 4.09 kcal/mol). The observed pattern of total binding free energies for the various complexes suggested that the GyrA double mutation S83L-D87N might cause localised distortions in the structure of GyrA that diminish the van der Waals and electrostatic interactions, resulting in diminished binding affinity. On the other hand, the ParC mutant complex retained nearly equivalent energetic favourability to its wild-type counterpart, suggesting a very limited effect of the S80I substitution on ligand binding. To provide additional evidence to support these conclusions, a more extensive MM/PBSA study was performed using the Poisson-Boltzmann solvation model for a more detailed representation of electrostatics and solvent interactions (Hou et al., 2011). The total binding free energies obtained using the MM/PBSA methodology followed a trend similar to that of the MM/GBSA data. The total binding free energy for the GyrA_WT-CA_1617 complex was −13.82 ± 3.77 kcal/mol, which represented the lowest total binding energy of the complexes tested, followed by the GyrA_S83L_D87N mutant complex (−14.31 ± 4.62 kcal/mol). The total binding free energies for the ParC_WT-CA_1617 and ParC_S80I-CA_1617 complexes were −16.26 ± 3.76 kcal/mol and −16.69 ± 4.67 kcal/mol, respectively. The identical negative ΔG values for each complex obtained by both computational methodologies supported the notion that CA_1617 binds stably and spontaneously to each of the protein-ligand complexes tested.

**TABLE 11:** Binding free energy calculation of all the complexes through MM/GBSA method.

Complex	ΔVDWAALS*	ΔEEL*	ΔEGB*	ΔESURF*	ΔGGAS*	ΔGSOLV*	ΔG_bind (total)*
GyrA_WT-CA_1617	−33.52 ± 4.90	−25.96 ± 7.15	43.57 ± 7.13	−4.45 ± 0.64	−59.47 ± 8.90	39.11 ± 6.99	−20.36 ± 4.87
GyrA_S83L_D87N-CA_1617	−27.64 ± 4.84	−17.59 ± 10.51	32.23 ± 8.90	−3.80 ± 0.71	−45.23 ± 11.04	28.43 ± 8.80	−16.81 ± 4.25
ParC_WT-CA_1617	−29.48 ± 4.31	−21.93 ± 7.93	37.28 ± 8.84	−3.93 ± 0.70	−51.41 ± 9.81	33.35 ± 8.37	−18.06 ± 3.17
ParC_S80I-CA_1617	−31.44 ± 4.47	−57.05 ± 16.70	73.21 ± 14.90	−4.74 ± 0.62	−88.49 ± 17.05	68.47 ± 14.66	−20.02 ± 4.09

* All units are reported in kcal/mol.

**TABLE 12:** Binding free energy calculation of all the complexes through MM/PBSA method.

Complex	ΔVDWAALS*	ΔEEL*	ΔEPB*	ΔENPOLAR*	ΔGGAS*	ΔGSOLV*	ΔG_bind (total)*
GyrA_WT-CA_1617	−33.36 ± 4.76	−25.97 ± 7.33	49.21 ± 8.02	−3.71 ± 0.39	−59.32 ± 8.93	45.5 ± 7.90	−13.82 ± 3.77
GyrA_S83L_D87N-CA_1617	−27.71 ± 4.97	−17.75 ± 10.57	34.36 ± 9.25	−3.22 ± 0.51	−45.45 ± 11.20	31.14 ± 9.18	−14.31 ± 4.62
ParC_WT-CA_1617	−29.51 ± 4.31	−22.04 ± 7.89	38.51 ± 10.09	−3.21 ± 0.54	−51.56 ± 9.94	35.3 ± 9.69	−16.26 ± 3.76
ParC_S80I-CA_1617	−31.41 ± 4.43	−57.04 ± 16.64	75.49 ± 15.33	−3.73 ± 0.35	−88.44 ± 17.03	71.76 ± 15.18	−16.69 ± 4.67

* All units are reported in kcal/mol.

The slightly higher solvation penalties observed with MM/PBSA compared to MM/GBSA (Tables 11, 12) arise from methodological differences rather than inconsistent binding behaviour. As MM/PBSA employs the Poisson-Boltzmann model, which treats solvent polarisation and dielectric effects more rigorously than the GB approximation, often yielding larger polar solvation energies for polar ligands like CA_1617. Although MM/PBSA yielded slightly higher solvation energy penalties than did MM/GBSA, the difference in binding free energy for CA_1617 was negligible, indicating that CA_1617 retains significant and highly specific interactions even under stringent solvation modelling. Both the methods consistently predicted favourable overall binding, indicating robust ligand recognition.

## 4 Discussion

Shigellosis, caused by the bacterium *Shigella*, continues to be one of the two leading causes of diarrhoea-related deaths worldwide, and can affect people of all ages, especially those living in poverty-stricken areas without adequate access to clean water and healthcare services (Hendrick et al., 2025). *Shigella flexneri* is the most frequent cause of gastrointestinal infections, and is most often found in developing countries, where it causes the most serious cases of disease. Unfortunately, in addition to being the most common source of disease, *S. flexneri* has emerged as a major concern because many of its strains have become resistant to multiple drugs, and many of these drug-resistant strains have developed resistance to the fluoroquinolones, the class of antibiotics commonly used to treat infections caused by bacteria like *Shigella* (Khalil et al., 2018). As a result of the emergence of drug-resistant *S. flexneri*, recent reports of community-based outbreaks of MDR *Shigella*, especially emerged among gay men who have sex with other men (MSM) in large cities, indicating a concerning shift in transmission dynamics and resistance evolution (O’Flanagan et al., 2023; Thorley et al., 2023).

The increasing number of emerging fluoroquinolone-resistant *S. flexneri* strains creates a significant challenge in terms of therapeutic options. QRDR-associated mutations in gyrA and parC reduce the binding efficiency of fluoroquinolones to their target enzymes, thereby compromising therapeutic effectiveness. The increasing prevalence of such resistant *S. flexneri* strains underscores the urgent need for novel inhibitors capable of targeting both DNA gyrase and topoisomerase IV, including their mutant variants. Developing agents that overcome this mutation-driven resistance mechanism is essential not only for improving treatment outcomes in shigellosis but also for limiting the spread of resistant strains under antibiotic selection pressure. In this context, the present study employed a machine-learning–guided, multi-tiered computational strategy to identify potential dual inhibitors of GyrA and ParC using MIC-based activity data and a curated library of ciprofloxacin analogues retrieved from the PubChem database. The significant correlation between the experimental and predicted activities indicates the effectiveness of ML-based models to prioritise compounds with structural viability for experimental testing. Among the models tested, the Random Forest-based model had the best predictive accuracy, and the lowest error rate, demonstrating the highest level of reliability, and the greatest degree of sensitivity and precision in distinguishing between active and inactive analogues. The predictive nature of this method allowed us to rapidly evaluate and prioritise structurally viable compounds with a high probability of displaying antibacterial activity. The subsequent evaluations of the candidate molecules using antibacterial assays, toxicology filters, DFT-based geometry optimisations, molecular docking, molecular dynamics simulations, PCA, and free energy of binding analysis formed a comprehensive evaluation of the potential of the candidate molecules to act as effective pharmaceuticals and to display stable binding to GyrA and ParC. Docking studies demonstrate that many of the analogues identified through the ML based screening exhibit greater predicted binding affinity to both GyrA and ParC than the control antibiotic ciprofloxacin. The compounds exhibit strong and stable non-covalent hydrogen bonding to conserved residues within the QRDR of GyrA and ParC, and some of the compounds exhibit hydrogen bonding to the same type of mutated residues as ciprofloxacin. After a series of evaluations, among many of the compounds, CA_1617 was found to remain compatible with the resistance altered active site of GyrA and ParC. The enhanced binding energy and increased interaction density of the derivatives support the hypothesis that the CA_1617 molecule can act as a dual-site inhibitor, which would likely reduce the likelihood of resistance development as a result of a mutation.

The MD simulations demonstrated that the RMSD trajectories remained consistent in terms of structure over the duration of the simulation, while the RMSF and Rg analyses illustrated increased compactness and reduced mobility of the catalytic region upon ligand binding. The consistent patterns of hydrogen bonding between the ligand and protein also suggest that the ligands maintain their position within the binding pocket, even when resistance-associated mutations are present. Therefore, the results of the MD simulations illustrate that the CA_1617 molecule exhibits a robust, energetically favourable fit in the binding pocket of both GyrA and ParC, suggesting that the molecule will retain its structural integrity under physiologic conditions.

The PCA and FEL analysis illustrate the importance of CA_1617 in affecting the protein’s flexibility and enabling the stabilisation of the global conformational architecture of both GyrA and ParC. The decrease in the conformational entropy, and the reduction of the size of the conformational space that the CA_1617 molecule when it binds to the protein, indicate that ligand binding causes an increase in the rigidity of the protein structure which is a common characteristic of functional complexes. The differences in the dynamics of the wild type and mutant GyrA provide critical insight into the effects of mutations that confer resistance. The S83L_D87N double substitution in GyrA produced a moderate level of flexibility and significantly reduced the homogeneity of the conformational sampling, consistent with previous studies indicating that these substitutions disrupt the shape of the quinolone-binding pocket and significantly reduce the affinity for inhibitors. The fact that CA_1617 is able to produce localised energy minima in the mutant system demonstrates its structural adaptability and its ability to recover from mutations that would cause instability to the protein. In contrast, the S80I substitution in ParC did not significantly affect the conformational sampling of the enzyme and CA_1617 bound to the wild-type and mutant forms of ParC equally well. The fact that CA_1617 maintains binding efficiency and structural integrity to the same degree on the wild-type and mutated forms of ParC indicates that the binding patterns of the ligands can be relatively insensitive to slight changes in the QRDR region of the enzyme. The results also suggest that CA_1617 effectively limits large-scale collective motions of both target enzymes by stabilising the structural core of the enzymes, thus preventing motions that may compromise the active state of the protein. The ability of the ligand to limit the conformational freedom of the protein, yet still allow for the necessary flexibility, will contribute to its dual inhibitory activity against GyrA and ParC. Together, the use of both MM/GBSA and MM/PBSA to analyse the molecular basis for the binding of CA_1617 to GyrA, ParC, and the mutant forms of these proteins provided additional information on the thermodynamics of the protein-ligand complex. Consistent with the trends for the dynamical stability of the complexes, both methods indicated that CA_1617 formed thermodynamically stable complexes with favourable binding energies relative to each of the wild-type and mutant enzymes.

Further, the energetic decomposition of the binding energies indicated that van der Waals and electrostatic interactions were the primary contributors to the stabilisation of the complexes, while the contributions from the polar solvation energies were relatively smaller. The relatively high polar solvation penalties observed in MM/PBSA analysis reflect the energetic cost of ligand desolvation prior to binding. This desolvation requirement may moderately influence association kinetics by requiring partial dehydration of both the ligand and catalytic pocket. However, once bound, favourable electrostatic and van der Waals interactions stabilise the complex and support sustained residence time. Thus, the increased potency of CA_1617 relative to its predecessor compounds appears to result from increased hydrophobic and electrostatic complementarity within the active site of the enzyme, a property that should facilitate recovery from the conformational changes induced by resistance mutations. Finally, the combined use of MM/GBSA and MM/PBSA provided rigorous thermodynamic validation of the binding strength of CA_1617 to GyrA, ParC, and their mutant forms, further supporting the potential for CA_1617 as a dual-target inhibitor of these enzymes. Through extensive computational evaluations, a small molecule scaffold that possesses strong dual-target affinity and acceptable pharmacokinetics was identified, suggesting the potential for this scaffold as a resilient fluoroquinolone derivative to resistant mutations. The identified compound, CA_1617 [PubChem CID: 13500666, IUPAC Name: 6-Fluoro-1-(4-fluorophenyl)-7-(4-methyl-3-oxopiperazin-1-yl)-4-oxoquinoline-3-carboxylic acid] has been previously reported in the literature to exhibit potent antibacterial efficacy against several species of bacterial pathogens with MIC values of <0.1–1 μg/mL (Chu et al., 1985). For example, this compound has been shown to exhibit potent antibacterial activity against several strains of *E. coli* juhl (MIC value 0.39 μg/mL), *Klebsiella pneumoniae* 8045 (MIC value 0.2 μg/mL), *Enterobacter* aerogenes ATCC 13048 (MIC value 0.39 μg/mL), and multiple other Gram-negative and Gram-positive bacteria, as presented in Supplementary Material S2; Supplementary Table S4 (data on antibacterial activity of the compound against a variety of bacterial pathogens was obtained from the ChEMBL database). Thus, these results provide evidence regarding the antibacterial activity of this compound. Overall, this research demonstrates how ML-driven *in silico* approaches can accelerate the discovery of novel antimicrobial agents, especially at a time when traditional antibiotics are rapidly losing their effectiveness against WHO-designated high-priority pathogens such as *S. flexneri*.

## 5 Conclusion

The increased incidence of diarrheal infections with fluoroquinolone-resistant *S*. *flexneri* (which was classified as a priority drug-resistant bacteria by the WHO Bacterial Priority Pathogens List (2019)) presents an important challenge to worldwide antimicrobial stewardship. Additionally, the decreased ability of fluoroquinolone antibiotics to be used effectively is a result of the high frequency of chromosomally encoded point mutations in gyrA and parC in *S. flexneri* strains. This demonstrates an urgent need for the development of alternative treatments capable of addressing multiple resistance mechanisms. In this study, we developed an ML-guided computational approach to identify dual-functional inhibitory molecules against both GyrA and ParC, including their clinically relevant mutants, in *S. flexneri*. We present a multi-tiered computational strategy to discover dual-functional inhibitory molecules effective against both wild-type and QRDR-mutated forms of GyrA and ParC in fluoroquinolone-resistant *S. flexneri*. By utilising ML-derived activity predictions on a large ciprofloxacin analogue pool from PubChem through ADME filtering, virtual screening, molecular docking, MD simulations, and binding-free-energy analysis, we identified structurally robust and pharmacologically viable candidates capable of overcoming mutation-driven resistance. This study identified a ciprofloxacin analogue, CA_1617 [6-Fluoro-1-(4-fluorophenyl)-7-(4-methyl-3-oxopiperazin-1-yl)-4-oxoquinoline-3-carboxylic acid], as a potent dual inhibitor of GyrA and ParC, including the wild type and most frequently observed mutants of GyrA and ParC. It demonstrated strong dual-target affinity and mutation resilience, suggesting its potential as a viable lead compound for combating fluoroquinolone-resistant *S. flexneri*. These findings also underscore that ML-guided computational discovery can accelerate the identification of next-generation fluoroquinolone derivatives and provide a reproducible framework for developing novel therapeutics against fluoroquinolone-resistant *S. flexneri*. However, further *in vitro* and *in vivo* studies are essential to confirm its efficacy and clinical safety, thereby facilitating its advancement towards therapeutic applicability in clinics.

## Data Availability

The original contributions presented in the study are included in the article/Supplementary Material, further inquiries can be directed to the corresponding author.
