# Supplementary material for: A dual functional inhibitory molecule to combat GyrA-ParC and associated mutants in fluoroquinolone-resistant Shigella flexneri causing shigellosis: an ML guided and quantum simulation-based in silico approach
Source: Front Bioinform. 2026 Jun 10;6:1765216. doi: 10.3389/fbinf.2026.1765216 (PMC13291547; doi:10.3389/fbinf.2026.1765216)
Supplement: Supplementary file 3 [file Table2.docx]

**Supplementary File 2**

**Supplementary Section 1**

**Used Sequences details**

>4CKL _1|Chain A|DNA gyrase subunit A [Escherichia coli]

VGRALPDVRDGLKPVHRRVLYAMNVLGNDWNKAYKKSARVVGDVIGKYHPHGDSAVYDTIVRMAQPFSLRYMLVDGQGNFGSIDGDSAAAMRYTEIRLAKIAHELMADLEKETVDFVDNYDGTEKIPDVMPTKIPNLLVNGSSGIAVGMATNIPPHNLTEVINGCLAYIDDEDISIEGLMEHIPGPDFPTAAIINGRRGIEEAYRTGRGKVYIRARAEVEVDAKTGRETIIVHEIPYQVNKARLIEKIAELVKEKRVEGISALRDESDKDGMRIVIEVKRDAVGEVVLNNLYSQTQLQVSFGINMVALHHGQPKIMNLKDIIAAFVRHRREVVTRRTIFELRKARDRAHILEALAVALANIDPIIELIRHAPTPAEAKTALVANPWQLGNVAAMLERAGDDAARPEWLEPEFGVRDGLYYLTEQQAQAILDLRLQKLTGLEHEKLLDEYKELLDQIAELLRILGSADRLMEVIREELELVREQFGDKRRTET

>NP_708120.1 DNA gyrase subunit A [Shigella flexneri 2a str. 301]

MSDLAREITPVNIEEELKSSYLDYAMSVIVGRALPDVRDGLKPVHRRVLYAMNVLGNDWNKAYKKSARVVGDVIGKYHPHGDSAVYDTIVRMAQPFSLRYMLVDGQGNFGSIDGDSAAAMRYTEIRLAKIAHELMADLEKETVDFVDNYDGTEKIPDVMPTKIPNLLVNGSSGIAVGMATNIPPHNLTEVINGCLAYIDDEDISIEGLMEHIPGPDFPTAAIINGRRGIEEAYRTGRGKVYIRARAEVEVDAKTGRETIIVHEIPYQVNKARLIEKIAELVKEKRVEGISALRDESDKDGMRIVIEVKRDAVGEVVLNNLYSQTQLQVSFGINMVALHHGQPKIMNLKDIIAAFVRHRREVVTRRTIFELRKARDRAHILEALAVALANIDPIIELIRHAPTPAEAKTALVANPWQLGNVAAMLERAGDDAARPEWLEPEFGVRDGLYYLTEQQAQAILDLRLQKLTGLEHEKLLDEYKELLDQIAELLRILGSADRLMEVIREELELVREQFGDKRRTEITANSADINLEDLITQEDVVVTLSHQGYVKYQPLSEYEAQRRGGKGKSAARIKEEDFIDRLLVANTHDHILCFSSRGRVYSMKVYQLPEATRGARGRPIVNLLPLEQDERITAILPVTEFEEGVKVFMATANGTVKKTVLTEFNRLRTAGKVAIKLVDGDELIGVDLTSGEDEVMLFSAEGKVVRFKESSVRAMGCNTTGVRGIRLGEGDKVVSLIVPRGDGAILTATQNGYGKRTAVAEYPTKSRATKGVISIKVTERNGLVVGAVQVDDCDQIMMITDAGTLVRTRVSEISIVGRNTQGVILIRTAEDENVVGLQRVAEPVDEEDLDTIDGSAAEGDDEIAPEVDVDDEPEEE

**Alignment analysis results (Local pairwise alignment using EMBOSS Water)**

**
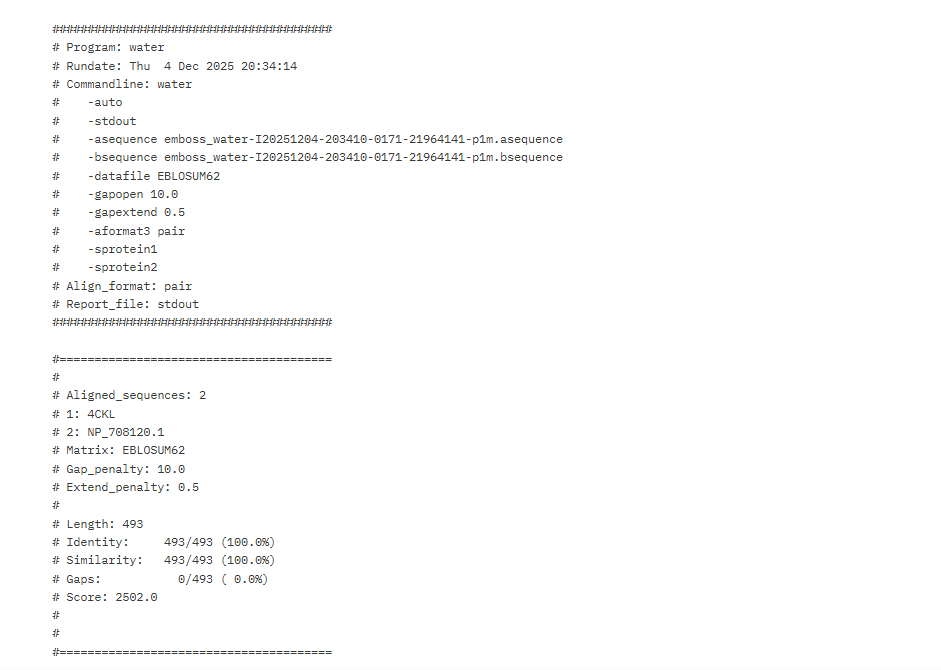
**

**
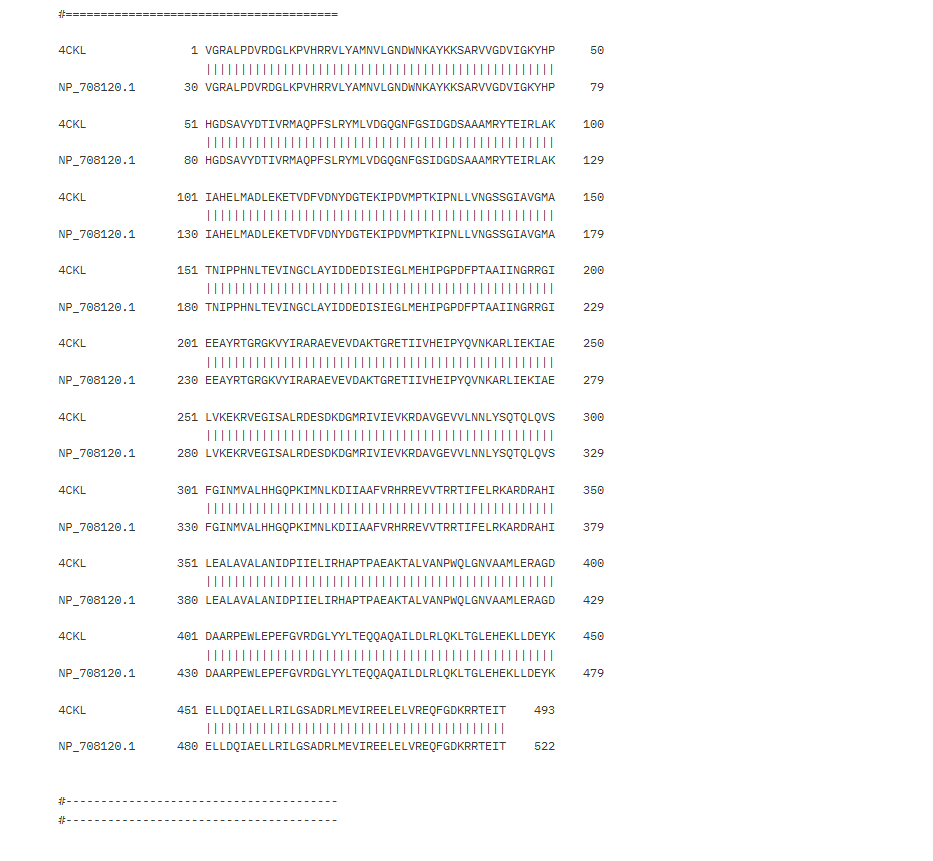
**

**Supplementary Figures**

**Supplementary Figure S1:** Protein Quality Validation Results of GyrA **(A)** and ParC **(B)**

1. **GyrA (Processed ): 4CKL**

**Overall model quality (Z-score: -9.24)**

**
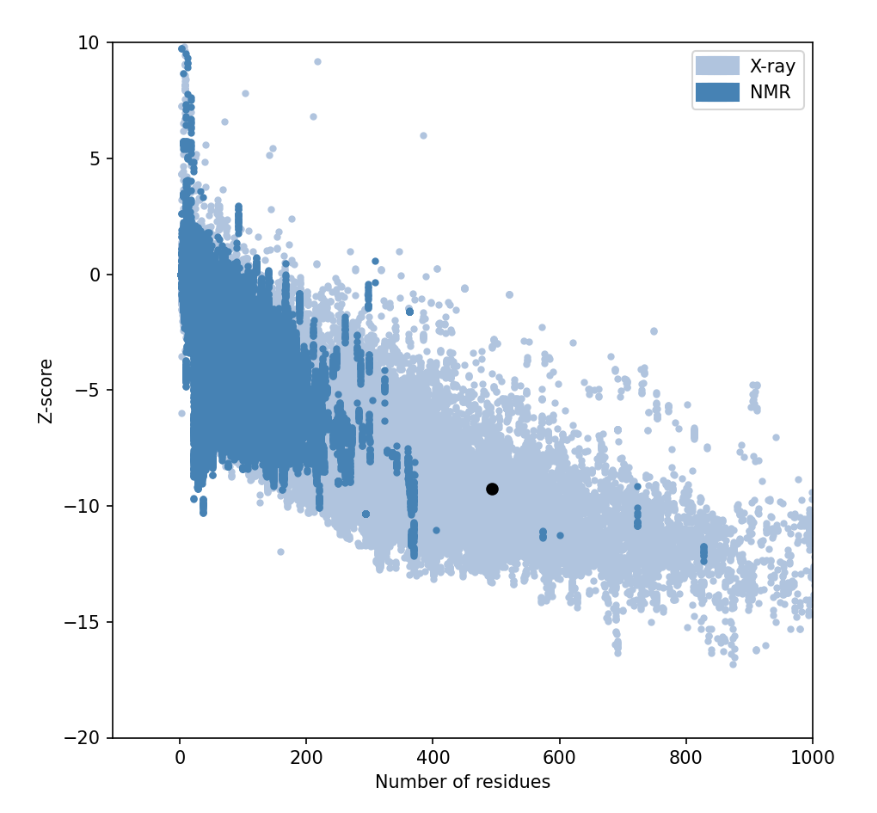
**

**Local model quality**

**
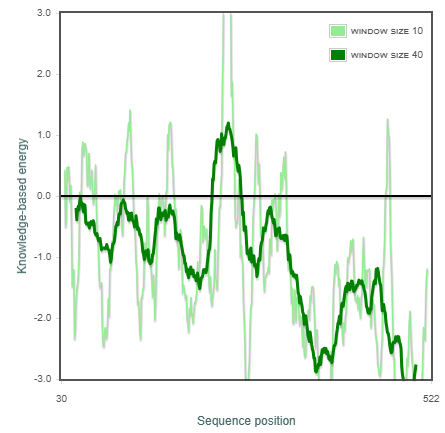
**

**Ramachandran Plot GyrA protein (PDB ID: 4CKL)**

**
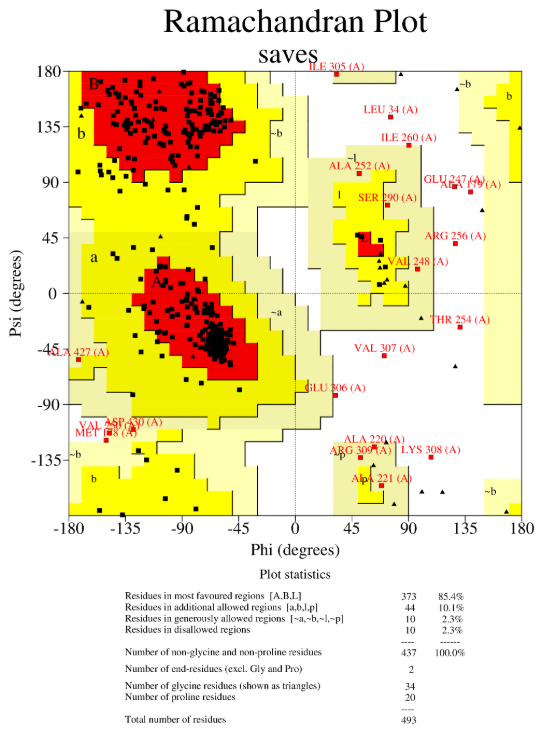
**

1. **ParC: AlphaFold-based structure (AlphaFold-based structure; ID: AF-P0AFI4-F1-v4)**

**Overall model quality (Z-Score: -9.34)**

**
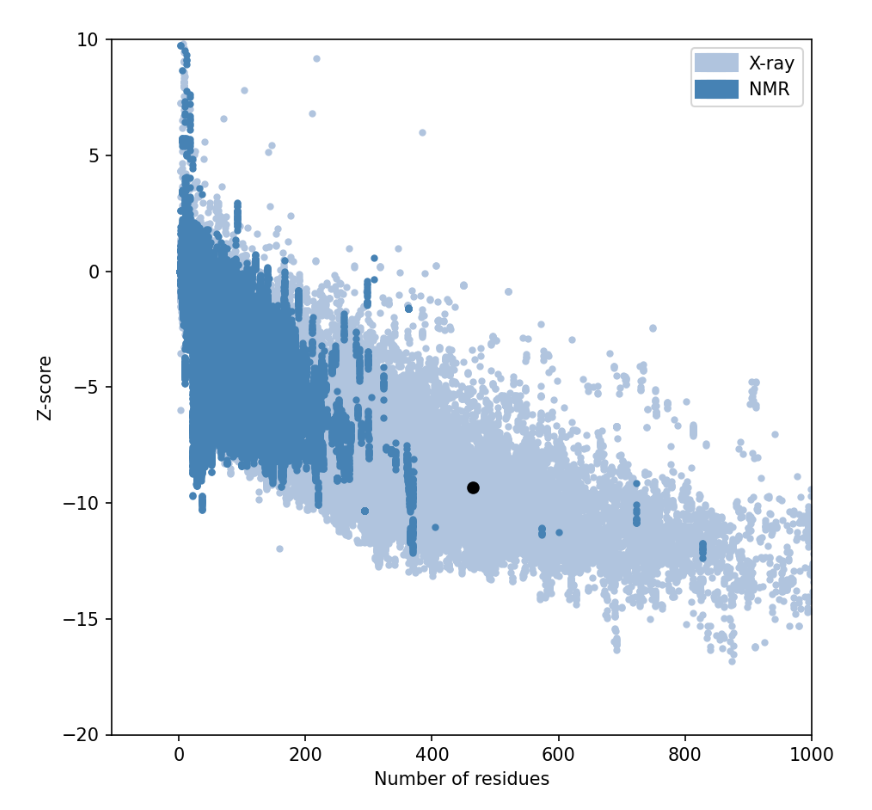
**

**Local model quality**

**
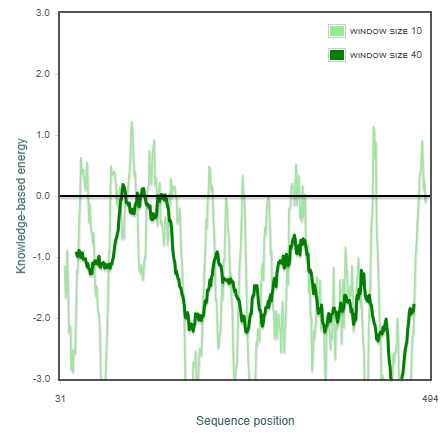
**

**Ramachandran Plot of ParC (AlphaFold-based structure; ID: AF-P0AFI4-F1-v4)
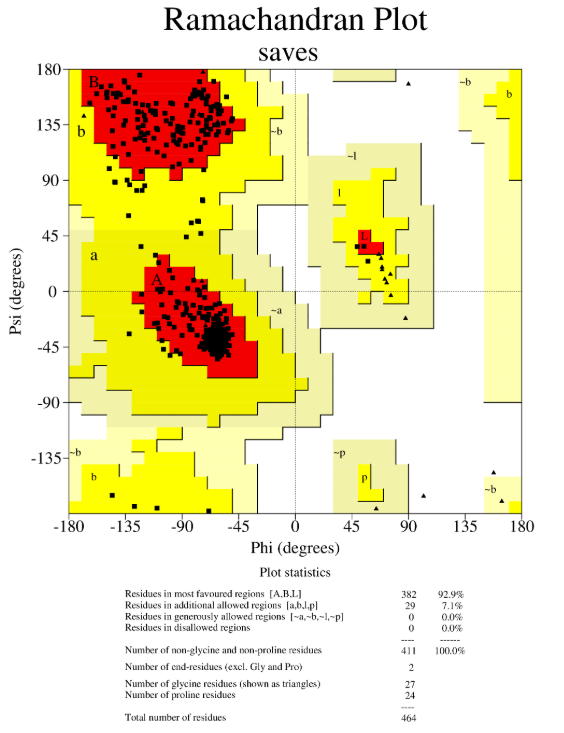
**

**Figure S2:** Interaction of DNA gyrase subunit A of *E.coli* (PDB ID: bound with gyrase inhibitor Simocyclinone D8


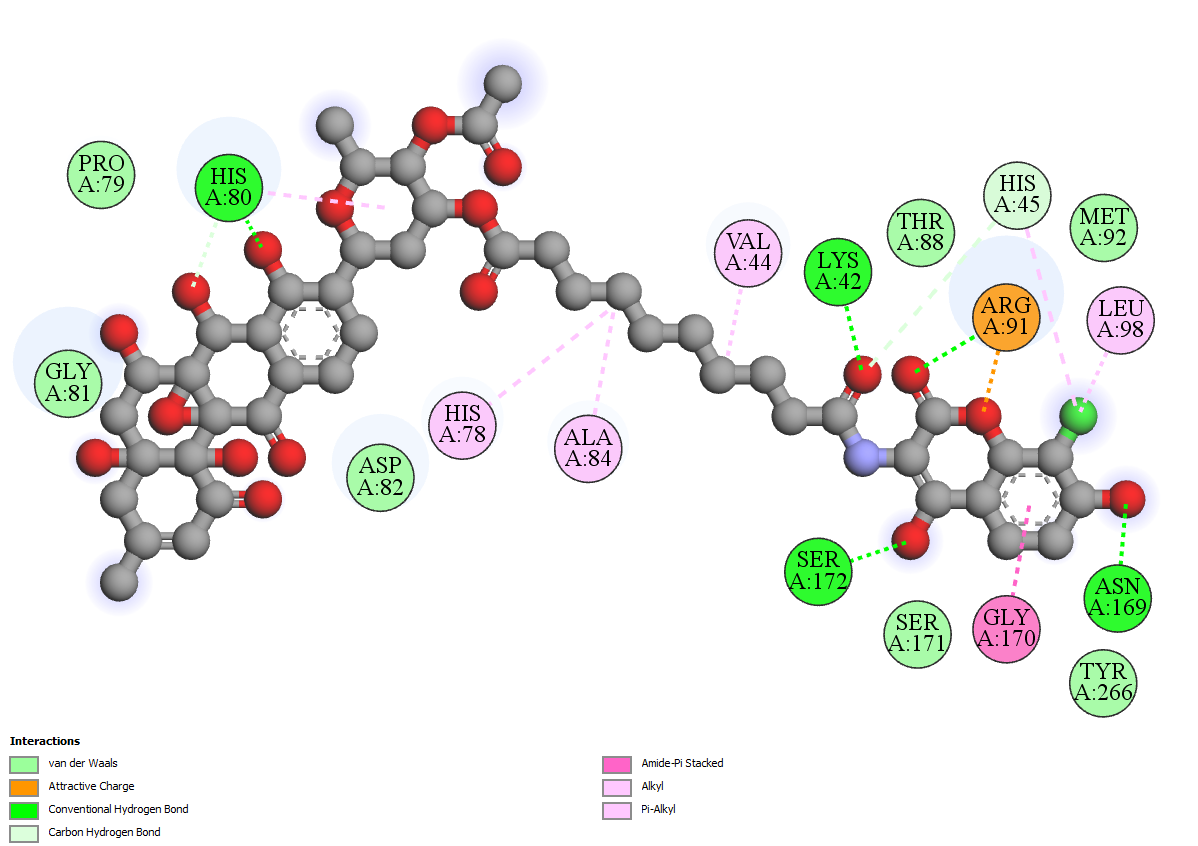


**Figure S3:** Root mean square fluctuations for the ligand upon binding with GyrA, ParC and their mutated proteins


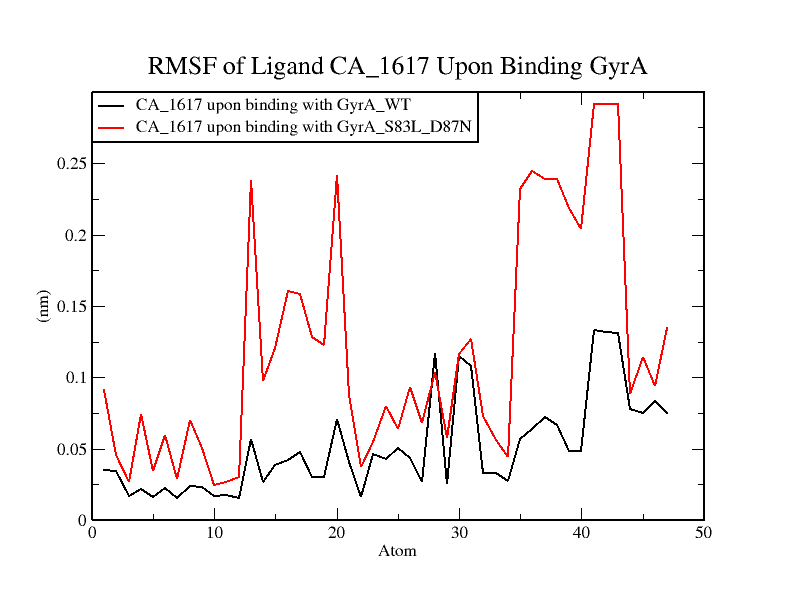


**
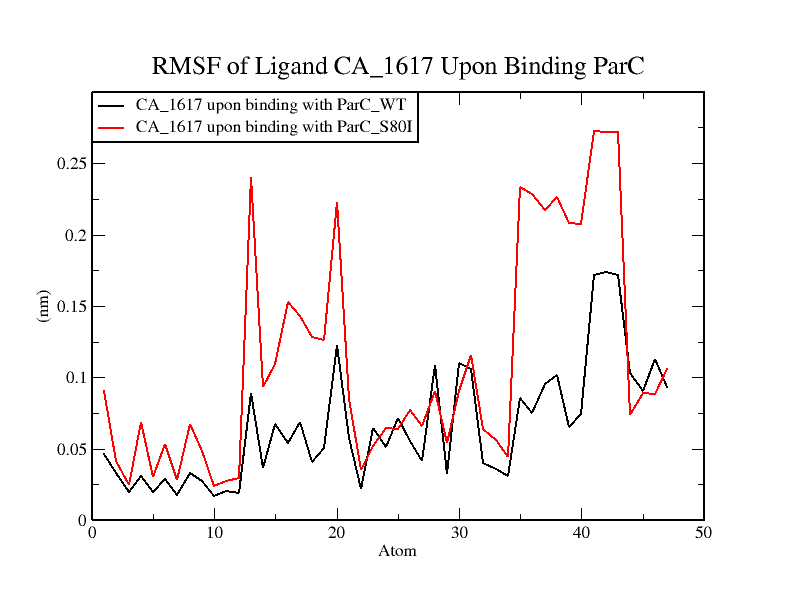
**

**Supplementary Tables**

**Table S1: List of descriptors**

| Sl. No. | **1D, 2D & 3D Descriptors and Fingerprints** | **Number** | **Descriptions of 1D, 2D & 3D Descriptors and Fingerprints** |
| --- | --- | --- | --- |
|  | **1D & 2D Descriptors** |  | **Descriptions** |
| 1 | Acidic Group Count | 1 | Number of acidic groups |
| 2 | ALOGP | 3 | ALOGP (Ghose-Crippen LogKow) and the Ghose-Crippen molar refractivity. |
| 3 | Apol | 1 | Sum of the atomic polarizabilities (including implicit hydrogens). |
| 4 | Aromatic Atoms Count | 1 | Number of aromatic atoms |
| 5 | Aromatic Bonds Count | 1 | Number of aromatic bonds |
| 6 | Atom Count | 14 | Total number of atoms and number of atoms of a certain element type |
| 7 | Autocorrelation | 346 | Autocorrelation descriptors (Moreau-Broto, Moran, Geary) |
| 8 | Barysz Matrix | 91 | Barysz matrix descriptors |
| 9 | Basic Group Count | 1 | Number of basic groups. The list of basic groups is defined by SMARTS originally presented in JOELib. |
| 10 | BCUT | 6 | Based on a weighted version of the Burden matrix, which takes into account both the connectivity as well as atomic properties of a molecule |
| 11 | Bond Count | 10 | Number of bonds of a certain bond order. |
| 12 | BPol | 1 | Sum of the absolute value of the difference between atomic polarizabilities of all bonded atoms in the molecule (including implicit hydrogens) |
| 13 | Burden Modified Eigenvalues | 96 | Burden modified eigenvalues |
| 14 | Carbon Types | 9 | Topological descriptor characterizing the carbon connectivity |
| 15 | Chi Chain | 10 | Simple and valence chi chain descriptors of orders 3, 4, 5, 6 and 7 |
| 16 | Chi Cluster | 8 | Simple and valence chi cluster descriptors of orders 3, 4,5 and 6 |
| 17 | Chi Path Cluster | 6 | Simple and valence chi path cluster descriptors of orders 3, 4,5 and 6 |
| 18 | Chi Path | 32 | Simple and valence chi path descriptors of orders 0 to 7 |
| 19 | Constitutional | 12 | Constitutional descriptors like sum/mean of atomic vDW, electronegativities, polarizabilities |
| 20 | Crippen logP and MR | 2 | Wildman-Crippen LogP and MR |
| 21 | Detour Matrix | 11 | Detour matrix descriptors |
| 22 | Eccentric Connectivity Index | 1 | A topological descriptor combining distance and adjacency information |
| 23 | Estate Atom Type | 489 | Electrotopological state descriptors |
| 24 | Extended Topochemical Atom | 43 | Extended Topochemical Atom (ETA) descriptors |
| 25 | FMF | 1 | Complexity of a molecule |
| 26 | Fragment Complexity | 1 | Complexity of a system. C=abs(B^2-A^2+A)+H/100, where C=complexity, A=number of non-hydrogen atoms, B=number of bonds and H=number of heteroatoms |
| 27 | HBond Acceptor Count | 4 | Number of hydrogen bond acceptors |
| 28 | HBond Donor Count | 2 | Number of hydrogen bond donors |
| 29 | Hybridization Ratio | 1 | Fraction of sp3 carbons to sp2 carbons |
| 30 | Information Content | 42 | Multigraph information content indices |
| 31 | Kappa Shape Indices | 3 | Kier and Hall kappa molecular shape indices compare the molecular graph with minimal and maximal molecular graphs and are intended to capture different aspects of molecular shape |
| 32 | Largest Chain | 1 | Number of atoms in the largest chain |
| 33 | Largest Pi System | 1 | Number of atoms in the largest pi system |
| 34 | Longest Aliphatic Chain | 1 | Number of atoms in the longest aliphatic chain |
| 35 | Mannhold LogP | 1 | Prediction of logP based on the number of carbon and hetero atoms |
| 36 | McGowan Volume | 1 | McGowan characteristic volume |
| 37 | MDE | 19 | Molecular Distance Edge descriptors |
| 38 | MLFER | 6 | Molecular linear free energy relation |
| 39 | Path Count | 22 | Path counts |
| 40 | Petitjean Number | 1 | Eccentricity of a vertex corresponds to the distance from that vertex to the most remote vertex in the graph |
| 41 | Ring Count | 68 | Number of ring |
| 42 | Rotatable Bonds Count | 4 | Number of rotatable bond |
| 43 | Rule Of Five | 1 | Number of failures of the Lipinski's Rule Of 5 |
| 44 | Topological | 3 | Topological descriptors, such as topological radius, topological diameter and topological shape |
| 45 | Topological Charge | 21 | Topological charge descriptors |
| 46 | Topological Distance Matrix | 11 | Topological distance matrix descriptors |
| 47 | TPSA | 1 | Topological polar surface area based on fragment contributions (TPSA) |
| 48 | VABC | 1 | Van der Waals volume calculated using the method proposed in [Zhao, Yuan H. and Abraham, Michael H. and Zissimos, Andreas M., Fast Calculation of van der Waals Volume as a Sum of Atomic and Bond Contributions and Its Application to Drug Compounds, The Journal of Organic Chemistry, 2003, 68:7368-7373]. |
| 49 | VAdjMa | 1 | Vertex adjacency information (magnitude): 1 + log2 m where m is the number of heavy-heavy bonds. If m is zero, then zero is returned |
| 50 | Walk Count | 20 | Walk counts |
| 51 | Weight | 2 | Molecular weight |
| 52 | Weighted Path | 5 | Characterize molecular branching |
| 53 | Wiener Numbers | 2 | Wiener Path number and the Wiener Polarity Number |
| 54 | XLogP | 1 | Prediction of logP based on the atom-type method called XLogP |
| 55 | Zagreb Index | 1 | Sum of the squares of atom degree over all heavy atoms i |
|  |  |  |  |
|  | **3D Descriptors** |  | **Descriptions** |
| 1 | Autocorrelation3D | 80 | 3D topological distance based autocorrelation |
| 2 | CPSA | 29 | Charged Partial Surface Area (CPSA) descriptors, which are related to the Polar Surface Area descriptors |
| 3 | Gravitational Index | 9 | Characterize the mass distribution of the molecule |
| 4 | Length Over Breadth | 2 | Length over breadth descriptors |
| 5 | Moment Of Inertia | 7 | Moment of inertia and radius of gyration. Moment of inertia (MI) values characterize the mass distribution of a molecule |
| 6 | Petitjean Shape Index | 3 | Petitjean shape indices |
| 7 | RDF | 210 | Radial distribution function |
| 8 | WHIM | 91 | Weighted Holistic Invariant Molecular (WHIM) |
|  |  |  |  |
|  | **Fingerprints** |  | **Descriptions** |
| 1 | Pubchem Fingerprinter |  | Pubchem Fingerprinter |

**Table S2:** ADMET Evaluation of the top ten selected compounds

| **ADMET** | Compound Code | **CA_1432** | **CA_2177** | **CA_581** | **CA_2491** | **CA_2476** | **CA_1617** | **CA_2410** | **CA_83** | **CA_3218** | **CA_2908** |
| --- | --- | --- | --- | --- | --- | --- | --- | --- | --- | --- | --- |
|  | PubChem CIDs | 11796525 | 18329314 | 502070 | 44371371 | 44367335 | **13500666** | 44338492 | 384539 | 145976317 | 101619401 |
| **Absorption** | Human Intestinal Absorption | Absorbed (High Confidence) | Absorbed (High Confidence) | Absorbed (High Confidence) | Absorbed (High Confidence) | Absorbed (High Confidence) | **Absorbed (High Confidence)** | Absorbed (High Confidence) | Absorbed (High Confidence) | Absorbed (High Confidence) | Absorbed (High Confidence) |
|  | Human Oral Bioavailability 20% | Non-Bioavailable (Low Confidence) | Bioavailable (Medium Confidence) | Bioavailable (Medium Confidence) | Bioavailable (High Confidence) | Bioavailable (High Confidence) | **Bioavailable (Medium Confidence)** | Bioavailable (Medium Confidence) | Bioavailable (High Confidence) | Bioavailable (Medium Confidence) | Bioavailable (Medium Confidence) |
|  | Human Oral Bioavailability 50% | Non-Bioavailable (High Confidence) | Bioavailable (Low Confidence) | Non-Bioavailable (Low Confidence) | Bioavailable (Medium Confidence) | Bioavailable (Medium Confidence) | **Bioavailable (Medium Confidence)** | Bioavailable (Medium Confidence) | Bioavailable (Medium Confidence) | Bioavailable (Medium Confidence) | Bioavailable (High Confidence) |
|  | P-Glycoprotein Inhibitor | Non-Inhibitor (High Confidence) | Non-Inhibitor (High Confidence) | Non-Inhibitor (High Confidence) | Non-Inhibitor (High Confidence) | Non-Inhibitor (High Confidence) | **Non-Inhibitor (High Confidence)** | Non-Inhibitor (High Confidence) | Non-Inhibitor (High Confidence) | Non-Inhibitor (High Confidence) | Non-Inhibitor (High Confidence) |
|  | P-Glycoprotein Substrate | Substrate (Low Confidence) | Non-Substrate (Low Confidence) | Non-Substrate (Low Confidence) | Non-Substrate (Low Confidence) | Substrate (Low Confidence) | **Non-Substrate (Low Confidence)** | Substrate (Low Confidence) | Substrate (Low Confidence) | Non-Substrate (Medium Confidence) | Substrate (Low Confidence) |
| **Distribution** | Blood-Brain Barrier | Non-Penetrable (High Confidence) | Non-Penetrable (Low Confidence) | Non-Penetrable (High Confidence) | Non-Penetrable (High Confidence) | Non-Penetrable (High Confidence) | **Non-Penetrable (High Confidence)** | Non-Penetrable (High Confidence) | Non-Penetrable (High Confidence) | Non-Penetrable (High Confidence) | Non-Penetrable (High Confidence) |
|  | Plasma Protein Binding | 68.16 (Proper therapeutic index) | 84.76 (Proper therapeutic index) | 67.31 (Proper therapeutic index) | 84.19 (Proper therapeutic index) | 53.61 (Proper therapeutic index) | **79.36 (Proper therapeutic index)** | 69.4 (Proper therapeutic index) | 53.81 (Proper therapeutic index) | 24.49 (Proper therapeutic index) | 54.2 (Proper therapeutic index) |
|  | Steady State Volume of Distribution | 1.98 ( High) | 0.89 ( High) | 1.18 ( High) | 1.61 ( High) | 1.9 ( High) | **1 ( High)** | 1.6 ( High) | 1.67 ( High) | 0.91 ( High) | 1.41 ( High) |
| **Metabolism** | Breast Cancer Resistance Protein | Inhibitor (Low Confidence) | Inhibitor (Medium Confidence) | Non-Inhibitor (Low Confidence) | Non-Inhibitor (Medium Confidence) | Inhibitor (Low Confidence) | **Non-Inhibitor (Medium Confidence)** | Non-Inhibitor (Medium Confidence) | Non-Inhibitor (High Confidence) | Non-Inhibitor (High Confidence) | Non-Inhibitor (High Confidence) |
|  | CYP 1A2 Inhibitor | Non-Inhibitor (High Confidence) | Non-Inhibitor (High Confidence) | Non-Inhibitor (Medium Confidence) | Non-Inhibitor (High Confidence) | Non-Inhibitor (High Confidence) | **Non-Inhibitor (High Confidence)** | Non-Inhibitor (High Confidence) | Non-Inhibitor (High Confidence) | Non-Inhibitor (High Confidence) | Non-Inhibitor (High Confidence) |
|  | CYP 1A2 substrate | Non-Substrate (High Confidence) | Non-Substrate (High Confidence) | Non-Substrate (Medium Confidence) | Non-Substrate (High Confidence) | Non-Substrate (Medium Confidence) | **Non-Substrate (High Confidence)** | Non-Substrate (High Confidence) | Non-Substrate (High Confidence) | Non-Substrate (Low Confidence) | Non-Substrate (High Confidence) |
|  | CYP 2C19 Inhibitor | Non-Inhibitor (High Confidence) | Non-Inhibitor (High Confidence) | Non-Inhibitor (High Confidence) | Non-Inhibitor (High Confidence) | Non-Inhibitor (High Confidence) | **Non-Inhibitor (High Confidence)** | Non-Inhibitor (High Confidence) | Non-Inhibitor (High Confidence) | Non-Inhibitor (High Confidence) | Non-Inhibitor (High Confidence) |
|  | CYP 2C19 substrate | Non-Substrate (Medium Confidence) | Non-Substrate (Medium Confidence) | Non-Substrate (Low Confidence) | Non-Substrate (Low Confidence) | Non-Substrate (Low Confidence) | **Non-Substrate (Low Confidence)** | Non-Substrate (Medium Confidence) | Non-Substrate (Low Confidence) | Non-Substrate (Low Confidence) | Non-Substrate (Medium Confidence) |
|  | CYP 2C9 Inhibitor | Non-Inhibitor (High Confidence) | Inhibitor (Low Confidence) | Non-Inhibitor (High Confidence) | Non-Inhibitor (High Confidence) | Non-Inhibitor (High Confidence) | **Non-Inhibitor (Medium Confidence)** | Non-Inhibitor (High Confidence) | Non-Inhibitor (High Confidence) | Non-Inhibitor (High Confidence) | Non-Inhibitor (High Confidence) |
|  | CYP 2C9 Substrate | Non-Substrate (High Confidence) | Non-Substrate (High Confidence) | Non-Substrate (High Confidence) | Non-Substrate (High Confidence) | Non-Substrate (High Confidence) | **Non-Substrate (High Confidence)** | Non-Substrate (High Confidence) | Non-Substrate (High Confidence) | Non-Substrate (High Confidence) | Non-Substrate (High Confidence) |
|  | CYP 2D6 Inhibitor | Non-Inhibitor (High Confidence) | Non-Inhibitor (High Confidence) | Non-Inhibitor (High Confidence) | Non-Inhibitor (High Confidence) | Non-Inhibitor (High Confidence) | **Non-Inhibitor (High Confidence)** | Non-Inhibitor (High Confidence) | Non-Inhibitor (High Confidence) | Non-Inhibitor (High Confidence) | Non-Inhibitor (High Confidence) |
|  | CYP 2D6 Substrate | Non-Substrate (Medium Confidence) | Non-Substrate (Medium Confidence) | Substrate (Low Confidence) | Non-Substrate (High Confidence) | Non-Substrate (Low Confidence) | **Non-Substrate (High Confidence)** | Non-Substrate (High Confidence) | Non-Substrate (High Confidence) | Non-Substrate (High Confidence) | Non-Substrate (High Confidence) |
|  | CYP 3A4 Inhibitor | Non-Inhibitor (High Confidence) | Non-Inhibitor (High Confidence) | Non-Inhibitor (High Confidence) | Non-Inhibitor (High Confidence) | Non-Inhibitor (High Confidence) | **Non-Inhibitor (High Confidence)** | Non-Inhibitor (High Confidence) | Non-Inhibitor (High Confidence) | Non-Inhibitor (High Confidence) | Non-Inhibitor (High Confidence) |
|  | CYP 3A4 Substrate | Non-Substrate (High Confidence) | Non-Substrate (High Confidence) | Non-Substrate (High Confidence) | Non-Substrate (High Confidence) | Non-Substrate (Medium Confidence) | **Non-Substrate (High Confidence)** | Non-Substrate (High Confidence) | Non-Substrate (High Confidence) | Non-Substrate (High Confidence) | Non-Substrate (High Confidence) |
|  | OATP1B1 | Non-Inhibitor (Medium Confidence) | Non-Inhibitor (Low Confidence) | Non-Inhibitor (Medium Confidence) | Non-Inhibitor (Low Confidence) | Non-Inhibitor (Low Confidence) | **Non-Inhibitor (Low Confidence)** | Non-Inhibitor (Medium Confidence) | Non-Inhibitor (High Confidence) | Non-Inhibitor (High Confidence) | Non-Inhibitor (Low Confidence) |
|  | OATP1B3 | Non-Inhibitor (High Confidence) | Non-Inhibitor (High Confidence) | Non-Inhibitor (Medium Confidence) | Non-Inhibitor (Medium Confidence) | Non-Inhibitor (Medium Confidence) | **Non-Inhibitor (Medium Confidence)** | Non-Inhibitor (High Confidence) | Non-Inhibitor (High Confidence) | Non-Inhibitor (High Confidence) | Non-Inhibitor (High Confidence) |
| **Excretion** | Clearance | None | None | None | None | None | **None** | None | None | None | None |
|  | Organic Cation Transporter 2 | Non-Inhibitor (High Confidence) | Non-Inhibitor (High Confidence) | Non-Inhibitor (Medium Confidence) | Non-Inhibitor (High Confidence) | Non-Inhibitor (Medium Confidence) | **Non-Inhibitor (High Confidence)** | Non-Inhibitor (High Confidence) | Non-Inhibitor (High Confidence) | Non-Inhibitor (High Confidence) | Non-Inhibitor (High Confidence) |
| **Toxicity** | AMES Mutagenesis | Toxic (High Confidence) | Toxic (Low Confidence) | Safe (Low Confidence) | Toxic (High Confidence) | Toxic (Low Confidence) | **Safe (Low Confidence)** | Safe (High Confidence) | Toxic (Medium Confidence) | Toxic (High Confidence) | Toxic (High Confidence) |
|  | Avian | Safe (Medium Confidence) | Safe (High Confidence) | Safe (High Confidence) | Safe (Medium Confidence) | Safe (High Confidence) | **Safe (Medium Confidence)** | Safe (High Confidence) | Safe (High Confidence) | Safe (High Confidence) | Safe (High Confidence) |
|  | Bee | Safe (Medium Confidence) | Safe (Medium Confidence) | Safe (Low Confidence) | Safe (Low Confidence) | Toxic (High Confidence) | **Safe (Medium Confidence)** | Toxic (Medium Confidence) | Safe (Low Confidence) | Toxic (High Confidence) | Toxic (Medium Confidence) |
|  | Biodegradation | Safe (High Confidence) | Safe (High Confidence) | Safe (High Confidence) | Safe (High Confidence) | Safe (High Confidence) | **Safe (High Confidence)** | Safe (High Confidence) | Safe (High Confidence) | Safe (High Confidence) | Safe (High Confidence) |
|  | Carcinogenesis | Safe (High Confidence) | Safe (Low Confidence) | Safe (Medium Confidence) | Safe (High Confidence) | Safe (High Confidence) | **Safe (Medium Confidence)** | Safe (High Confidence) | Safe (Medium Confidence) | Safe (High Confidence) | Safe (High Confidence) |
|  | Liver Injury I (DILI) | Toxic (Medium Confidence) | Toxic (Medium Confidence) | Toxic (Low Confidence) | Toxic (Low Confidence) | Safe (Low Confidence) | **Safe (Low Confidence)** | Toxic (Low Confidence) | Toxic (Low Confidence) | Toxic (Low Confidence) | Toxic (Low Confidence) |
|  | Eye Corrosion | Safe (High Confidence) | Safe (High Confidence) | Safe (High Confidence) | Safe (High Confidence) | Safe (High Confidence) | **Safe (High Confidence)** | Safe (High Confidence) | Safe (High Confidence) | Safe (High Confidence) | Safe (High Confidence) |
|  | Eye irritation | Safe (High Confidence) | Safe (High Confidence) | Safe (High Confidence) | Safe (High Confidence) | Safe (High Confidence) | **Safe (High Confidence)** | Safe (High Confidence) | Safe (High Confidence) | Safe (High Confidence) | Safe (High Confidence) |
|  | Maximum Tolerated Dose | 0.87 (High) | 0.47 (High) | 0.35 (Low) | 0.44 (Low) | 0.32 (Low) | **0.61 (High)** | 0.13 (Low) | 0.8 (High) | 0.12 (Low) | 0.08 (Low) |
|  | hERG Blockers | Safe (High Confidence) | Safe (High Confidence) | Toxic (High Confidence) | Toxic (High Confidence) | Toxic (Low Confidence) | **Safe (High Confidence)** | Safe (High Confidence) | Safe (Medium Confidence) | Safe (High Confidence) | Toxic (High Confidence) |
|  | NR-AhR | Safe (High Confidence) | Safe (High Confidence) | Safe (High Confidence) | Safe (High Confidence) | Safe (High Confidence) | **Safe (High Confidence)** | Safe (High Confidence) | Safe (High Confidence) | Safe (High Confidence) | Safe (High Confidence) |
|  | NR-AR | Safe (High Confidence) | Safe (High Confidence) | Safe (Medium Confidence) | Safe (High Confidence) | Toxic (Medium Confidence) | **Safe (High Confidence)** | Safe (High Confidence) | Safe (High Confidence) | Safe (High Confidence) | Toxic (Medium Confidence) |
|  | NR-AR-LBD | Safe (High Confidence) | Safe (High Confidence) | Safe (High Confidence) | Safe (High Confidence) | Safe (High Confidence) | **Safe (High Confidence)** | Safe (High Confidence) | Safe (High Confidence) | Safe (High Confidence) | Toxic (Low Confidence) |
|  | NR-Aromatase | Safe (High Confidence) | Safe (High Confidence) | Safe (High Confidence) | Safe (High Confidence) | Safe (High Confidence) | **Safe (High Confidence)** | Safe (High Confidence) | Safe (High Confidence) | Safe (High Confidence) | Safe (High Confidence) |
|  | NR-ER | Safe (High Confidence) | Safe (High Confidence) | Safe (High Confidence) | Safe (High Confidence) | Safe (High Confidence) | **Safe (High Confidence)** | Safe (High Confidence) | Safe (High Confidence) | Safe (High Confidence) | Safe (Medium Confidence) |
|  | NR-ER-LBD | Safe (High Confidence) | Safe (High Confidence) | Safe (High Confidence) | Safe (High Confidence) | Safe (High Confidence) | **Safe (High Confidence)** | Safe (High Confidence) | Safe (High Confidence) | Safe (High Confidence) | Safe (High Confidence) |
|  | NR-GR | Safe (High Confidence) | Safe (High Confidence) | Safe (Medium Confidence) | Safe (Medium Confidence) | Safe (High Confidence) | **Safe (High Confidence)** | Safe (High Confidence) | Safe (High Confidence) | Safe (Medium Confidence) | Safe (Low Confidence) |
|  | NR-PPAR-gamma | Safe (High Confidence) | Safe (High Confidence) | Safe (High Confidence) | Safe (High Confidence) | Safe (High Confidence) | **Safe (High Confidence)** | Safe (High Confidence) | Safe (High Confidence) | Safe (High Confidence) | Safe (High Confidence) |
|  | NR-TR | Safe (High Confidence) | Safe (High Confidence) | Safe (High Confidence) | Safe (Medium Confidence) | Safe (High Confidence) | **Safe (High Confidence)** | Safe (High Confidence) | Safe (High Confidence) | Safe (High Confidence) | Safe (High Confidence) |
|  | Rat (Acute) | None | None | None | None | None | **None** | None | None | None | None |
|  | Rat (Chronic Oral) | None | None | None | None | None | **None** | None | None | None | None |
|  | Skin Sensitisation | Safe (Low Confidence) | Safe (Medium Confidence) | Safe (Low Confidence) | Safe (Medium Confidence) | Safe (Medium Confidence) | **Safe (Medium Confidence)** | Safe (Low Confidence) | Safe (Medium Confidence) | Toxic (Low Confidence) | Toxic (Low Confidence) |
|  | SR-ARE | Safe (Low Confidence) | Toxic (Medium Confidence) | Safe (High Confidence) | Safe (Medium Confidence) | Toxic (Low Confidence) | **Safe (Medium Confidence)** | Toxic (Medium Confidence) | Safe (High Confidence) | Safe (High Confidence) | Toxic (Medium Confidence) |
|  | SR-ATAD5 | Safe (High Confidence) | Safe (High Confidence) | Safe (High Confidence) | Safe (High Confidence) | Safe (High Confidence) | **Safe (High Confidence)** | Safe (High Confidence) | Safe (High Confidence) | Safe (High Confidence) | Safe (High Confidence) |
|  | SR-HSE | Safe (High Confidence) | Safe (High Confidence) | Safe (High Confidence) | Safe (High Confidence) | Safe (High Confidence) | **Safe (High Confidence)** | Safe (High Confidence) | Safe (High Confidence) | Safe (High Confidence) | Safe (High Confidence) |
|  | SR-MMP | Toxic (Low Confidence) | Safe (Medium Confidence) | Safe (High Confidence) | Safe (High Confidence) | Safe (High Confidence) | **Safe (High Confidence)** | Safe (High Confidence) | Safe (High Confidence) | Safe (High Confidence) | Safe (High Confidence) |
|  | SR-p53 | Safe (High Confidence) | Safe (High Confidence) | Safe (High Confidence) | Safe (High Confidence) | Safe (High Confidence) | **Safe (High Confidence)** | Safe (High Confidence) | Safe (High Confidence) | Safe (High Confidence) | Safe (High Confidence) |

**Table S3:** The interaction stability parameters of all proteins (GyrA, ParC and their selected mutants) complexes with CA_1617

|  | **Unit** | **Apo_GyrA_WT** | **GyrA_WT - CA_1617** | **Apo_GyrA_S83L_D87N** | **GyrA_S83L_D87N-CA_1617** | **Apo_ParC_WT** | **ParC_WT-CA_1617** | **Apo_ParC_S80I** | **ParC_S80I-CA_1617** |
| --- | --- | --- | --- | --- | --- | --- | --- | --- | --- |
| **RMSD** | **nm** | **0.44** | **0.539** | **0.418** | **0.612** | **0.197** | **0.227** | **0.237** | **0.302** |
| **RMSF** | **nm** | **0.185** | **0.272** | **0.167** | **0.191** | **0.133** | **0.153** | **0.152** | **0.146** |
| **Rg** | **nm** | **3.148** | **3.154** | **3.153** | **3.113** | **2.987** | **2.983** | **2.984** | **2.997** |
| **SASA** | **nm^2^** | **272.298** | **271.298** | **269.217** | **270.256** | **248.351** | **249.555** | **249.565** | **250.493** |
| **H bond** | **Max** |  | **3** |  | **5** |  | **4** |  | **4** |
|  | **Most Const.** |  | **2** |  | **2** |  | **2** |  | **2** |
| **Interaction energy** | **kJ/mol** |  | **-126.7** |  | **-103.509** |  | **-111.082** |  | **-117.259** |

**Table S4:** Antibacterial activity data retrieved from the ChEMBL Database

| Pubchem CID | Bacteria | MIC Value | | ChEMBL ID | Document ChEMBL ID |
| --- | --- | --- | --- | --- | --- |
| 1350066 | *Acinetobacter* sp CMX669 | 0.39 | ug.mL^-1^ | CHEMBL857905 | CHEMBL1122831 |
|  | *Streptococcus faecium* ATCC 8043 | 0.78 | ug.mL^-1^ | CHEMBL811834 |  |
|  | *Staphylococcus epidermidis* 3519 | 0.2 | ug.mL^-1^ | CHEMBL804396 |  |
|  | *Pseudomona aeruginosa* K799/WT | 0.78 | ug.mL^-1^ | CHEMBL763121 |  |
|  | *Klebsiella pneumoniae* 8045 | 0.2 | ug.mL^-1^ | CHEMBL704188 |  |
|  | *Escherichia coli* juhl | 0.39 | ug.mL^-1^ | CHEMBL676922 |  |
|  | *Staphylococcus aureus* ATCC 6538P | 0.1 | ug.mL^-1^ | CHEMBL804633 |  |
|  | *Pseudomona aeruginosa* 5007 | 1.56 | ug.mL^-1^ | CHEMBL763120 |  |
|  | *Staphylococcus aureus* CMX 686B | 0.2 | ug.mL^-1^ | CHEMBL811169 |  |
|  | *Streptococcus pyogenes* 930 | 0.39 | ug.mL^-1^ | CHEMBL811010 |  |
|  | *Enterobacter aerogenes* (*Klebsiella aerogens*) ATCC 13048 | 0.39 | ug.mL^-1^ | CHEMBL679099 |  |
